# IL-1**β** signaling modulates T follicular helper and regulatory cells in human lymphoid tissues

**DOI:** 10.1172/jci.insight.188724

**Published:** 2025-05-20

**Authors:** Romain Vaineau, Raphaël Jeger-Madiot, Samir Ali-Moussa, Laura Prudhomme, Hippolyte Debarnot, Nicolas Coatnoan, Johanna Dubois, Marie Binvignat, Hélène Vantomme, Bruno Gouritin, Gwladys Fourcade, Paul Engeroff, Aude Belbézier, Romain Luscan, Françoise Denoyelle, Roberta Lorenzon, Claire Ribet, Michelle Rosenzwajg, Bertrand Bellier, David Klatzmann, Nicolas Tchitchek, Stéphanie Graff-Dubois

**Affiliations:** 1 Immunology, Immunopathology, Immunotherapy, INSERM U959, Pitié Salpétrière Hospital, Sorbonne University, Paris, France.; 2 AP-HP, Hôpital Pitié-Salpêtrière, Clinical Investigation Center for Biotherapies (CIC-BTi) and Immunology-Inflammation-Infectiology and Dermatology Department (3iD), Paris, France.; 3 Department of Rheumatology, Saint-Antoine Hospital, Centre de Recherche Saint-Antoine, Paris Inserm UMRS 938, Sorbonne Université, Assistance Publique-Hôpitaux de Paris, Paris, France.; 4 Department of Immunology, University Clinic for Rheumatology and Immunology, University of Bern, Bern, Switzerland.; 5 Department of Pediatric Otolaryngology, AP-HP, Hôpital Necker-Enfants Malades, Paris, France.

**Keywords:** Autoimmunity, Immunology, Autoimmune diseases, Cellular immune response, T cells

## Abstract

Dysregulation of T follicular helper (Tfh) and T follicular regulatory (Tfr) cell homeostasis in germinal centers (GCs) can lead to antibody-mediated autoimmunity. While IL-1β modulates the GC response via IL-1R1 and IL-1R2 receptors on follicular T cells in animal models, its role in humans remains unclear. We analyzed Tfh and Tfr phenotypes in human secondary lymphoid organs (tonsils, spleen, and mesenteric lymph nodes) using flow cytometry, single-cell transcriptomics, and in vitro culture, comparing findings with samples from autoimmune patients. We observed organ-specific Tfh/Tfr phenotypes according to activation status and IL-1 receptor expression. An excess of IL-1R1 over IL-1R2 expression promoted a unique activated Tfr subset with Treg and GC-Tfh features. IL-1β signaling via IL-1R1 enhanced follicular T cell activation and Tfh-to-Tfr differentiation, while IL-1β inhibition upregulated IL-1R1, indicating a tightly regulated process. In autoimmune patients, high IL-1β and circulating Tfr levels correlated with increased autoantibody production, linking inflammation, IL-1β signaling, and Tfr/Tfh balance. Our findings highlight the critical role of IL-1β in follicular T cell activation and suggest that targeting IL-1β signaling in Tfh and Tfr cells could be a promising strategy for treating antibody-mediated autoimmune diseases.

## Abstract 2

This study explores how IL-1β signaling affects T follicular helper and regulatory cell activation in human lymphoid organs, potentially influencing autoimmune disease mechanisms.

## Graphical abstract

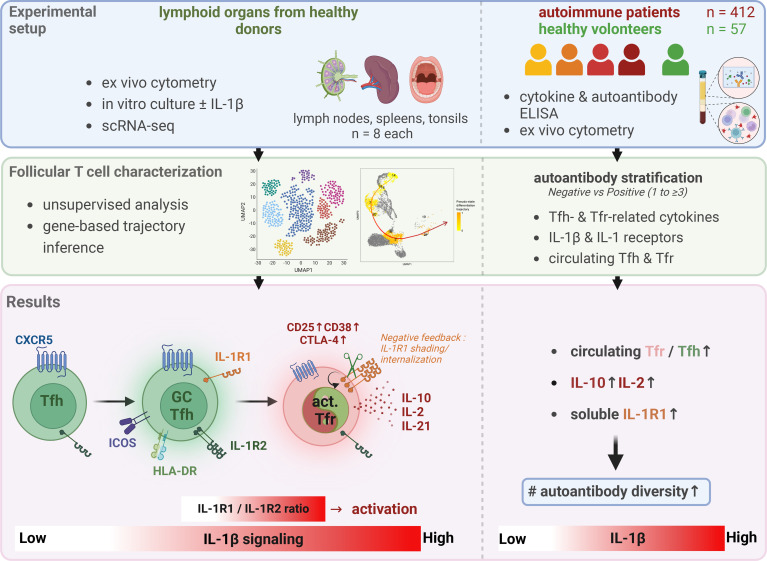

## Introduction

Germinal centers (GCs) are specialized structures enabling the production of high-affinity antibodies by B cells within secondary lymphoid organs (SLOs) (1–3). The GC reaction occurs following antigen-specific recognition, leading to the expansion of follicular helper T cells (Tfh) that support the maturation of cognate B cells (4–6). Excessive help provided during the GC reaction can lead to pathologic antibody production, notably in the context of autoimmune diseases (7). To maintain immune homeostasis, distinct regulatory mechanisms exist, encompassing external regulation via antibody feedback (8) or internal regulation through follicular regulatory T cells (Tfr) (9).

Tfr are a suppressive population derived from thymic Tregs (10), which exert their functions by direct inhibition or by the secretion of immunomodulatory cytokines (11–13). Accordingly, decreased circulating Tfr (cTfr) in the blood of patients are associated with disease onset and outcome in rheumatoid arthritis, systemic lupus erythematosus, and multiple sclerosis (14–16). However, emerging questions arise concerning the dichotomic view of Tfh and Tfr, by highlighting Tfr heterogeneity (17–19) or by pointing out Tfh as precursors of Tfr (20–22). Cytokines have a central role in the function of Tfh and Tfr, either by polarizing their differentiation (7) or by guiding their migration, such as by CXCL13 (23), or else by serving as effector cytokines — i.e. IL-21 and IL-10 for Tfh and Tfr, respectively (24, 25).

IL-1β is a proinflammatory cytokine with established roles in immune regulation, spanning both innate and adaptive responses (26). Indeed, IL-1β has been shown to enable T-dependent antibody responses (27), and to directly activate CD4^+^ T cells (28). While the impact of IL-1β in various inflammatory processes is acknowledged, its interaction with follicular T cells (Tfol) and subsequent GC reaction is not fully understood. Notably, the expression of IL-1 receptors (IL-1Rs) including IL-1R1, the agonist, and IL-1R2, the decoy, have been associated with follicular T cell function. In mice, both binding to and inhibition of IL-1R1 impact Tfh activation and functions (29–33). In particular, we previously demonstrated that the expression of IL-1R2 on murine Tfr cells inhibits IL-1R1–mediated activation of both Tfh and Tfr cells (32). In humans, it has been documented that cTfh are among the most responsive to IL-1β via NF-κB signaling (34).

Given the limited accessibility of human lymphoid organs, most studies on the reactivity of Tfh and Tfr cells to IL-1β have been confined to murine studies or to circulating human Tfh and Tfr. In the present study, we aimed to understand the contribution of IL-1β to the activation profile of healthy human lymphoid-resident Tfh and Tfr. We also seek to decipher the role of IL-1β in the context of pathological antibody production.

## Results

### Human secondary lymphoid organs differ in their follicular T cell composition and phenotype.

**Figure 1:**
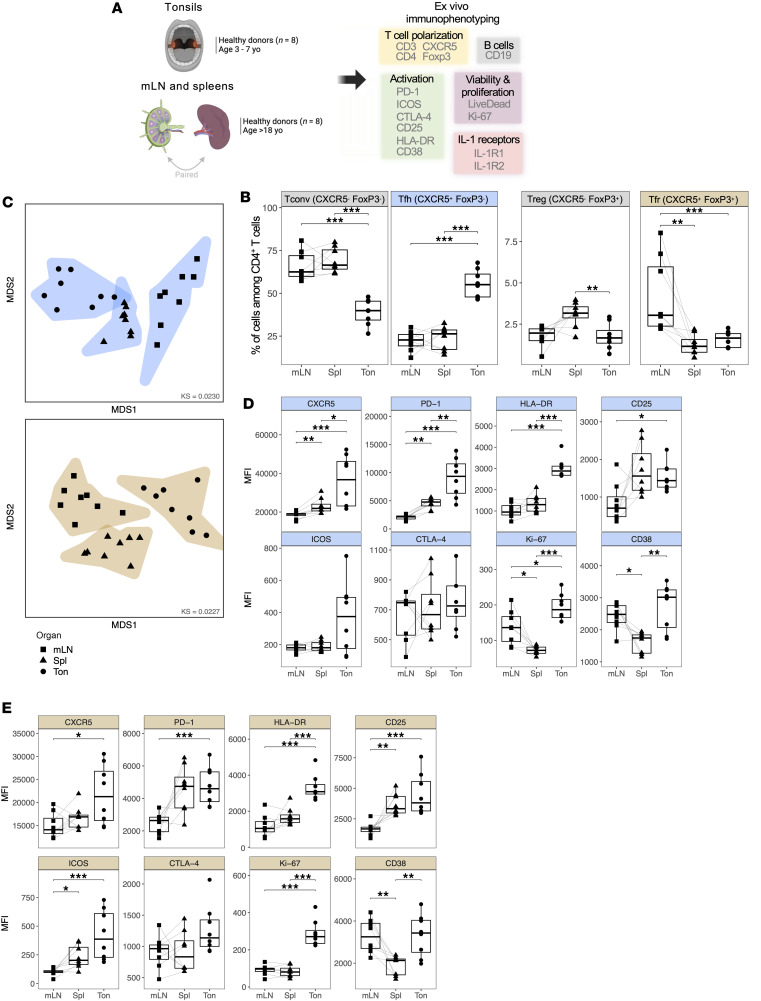
The abundance and phenotype of Tfol vary upon secondary lymphoid organs. (**A**) Experimental design for the immunophenotyping of cell suspensions extracted from healthy pediatric tonsils (*n* = 8), paired mesenteric lymph nodes (mLN, *n* = 8), and spleens (*n* = 8) from adult healthy donors. (**B**) Percentages of effector CXCR5^–^ Foxp3^–^ (Tconv) and CXCR5^+^ Foxp3^–^ (Tfh) cells, and regulatory CXCR5^–^ Foxp3^+^ (Treg) and CXCR5^+^ Foxp3^+^ (Tfr) cells among CD4^+^ T cells across lymphoid organs (*n* = 8 each). (**C**) Multidimensional scaling (MDS) plot displaying the similarities and dissimilarities between samples based on the MFI of all markers within Tfh (top, blue) and Tfr (bottom, gold) per sample, grouped by organ. (**D** and **E**) Mean fluorescence intensity (MFI) of markers of interest within Tfh (**D**) and Tfr (**E**) across lymphoid organs (*n* = 8 each). KS, Kruskal’s Stress. In **B**, **D**, and **E**, comparisons between mLN and Spleen have been assessed using a paired Wilcoxon test and comparisons between mLN and Spleen or Spleen and Tonsils have been assessed using a Mann-Whitney test. The FDR procedure has been applied for *P* value adjustments. *P* values are reported as the following: *P < 0.05; **P < 0.01; ***P < 0.001.

To characterize Tfh and Tfr among major CD4^+^ T cell subsets from different SLOs, we collected 8 pediatric tonsils (Ton), as well as 8 paired spleens (Spl) and mesenteric lymph nodes (mLNs) from unrelated healthy donors (Figure 1A). We hypothesized that these tissues would cover a wide range of Tfh and Tfr activation states, since they represent 3 types of SLOs: mucosa-associated, blood-draining, and lymph-draining organs, respectively. Using flow cytometry, we identified 4 major CD4^+^ T cell subsets based on CXCR5 and Foxp3 expression: Tfh and Tfr within Tfol, Tconv, and Treg within non-Tfol (Supplemental Figure 1A; supplemental material available online with this article; https://doi.org/10.1172/jci.insight.188724DS1).

The ex vivo abundance of Tfh among CD4^+^ T cells was significantly higher in tonsils than in spleen and mLNs (*P* < 0.001), as opposed to Tconv abundance which was decreased in tonsils (*P* < 0.001) (Figure 1B). The abundance of Treg differed in spleens with significantly increased frequency compared with tonsils (*P* < 0.01), while Tfr frequency was enriched in mLNs compared with spleens (*P* < 0.01) and tonsils (*P* < 0.001).

To address differences in Tfh and Tfr phenotypes between lymphoid organs, we integrated the expression of all markers by performing multidimensional scaling (MDS) on each population (Figure 1C). In Tfh (blue) and Tfr (brown), samples clustered separately and distinctly according to their organ of origin. Tfh and Tfr formed a gradient along the MDS1 dimension from tonsils to mLNs, aligning with the gradient of activation marker expressions.

We examined the phenotype of Tfh and Tfr across lymphoid organs by comparing the mean fluorescence intensity (MFI) of 8 markers related to cell activation (CXCR5, PD-1, HLA-DR, CD25, ICOS, and CD38), proliferation (Ki-67), and function (CTLA-4) (Supplemental Figure 1B). In Tfh, we observed a significant and gradual increase in the MFI of CXCR5, PD-1, HLA-DR, and CD25 within spleens and tonsils, compared with mLNs. ICOS and CTLA-4 were also increased following the same pattern, though not reaching significance (Figure 1D). Interestingly, both Ki-67 and CD38 MFI were significantly higher in tonsillar Tfh, whereas their expression was downregulated in the spleen compared with paired mLNs, suggesting a lack of activated and proliferating splenic Tfh. Tfh heterogeneity was observed regarding bimodal expression patterns of CXCR5, PD-1, and CD25, indicating variations in Tfh activation between organs and between donors (Supplemental Figure 1C).

Similarly, Tfr displayed a significant and gradual increase in the MFI of activation markers (CXCR5, PD-1, HLA-DR, CD25, and ICOS) in tonsils (Figure 1E), contrasting with its lower abundance in this organ. Ki-67 and CD38 expression in Tfr mirrored the expression of these markers in Tfh, underlining the higher proliferative and activated phenotype of tonsillar Tfh and Tfr compared with splenic counterparts. The Tfr population exhibited heterogeneity regarding the expression pattern of CXCR5, Foxp3, PD-1, CD25, and CTLA-4, indicating distinct phenotypes of Tfr within lymphoid organs (Supplemental Figure 1C).

In summary, our results show that the frequencies and phenotypes of Tfh and Tfr vary according to their SLO origin, suggesting a potential imprint of the SLO on follicular T cell biology and highlighting pediatric tonsils as the SLO with the most activated Tfol.

### The expression of IL-1 receptors differs between Tfh and Tfr according to lymphoid cell activation.

**Figure 2:**
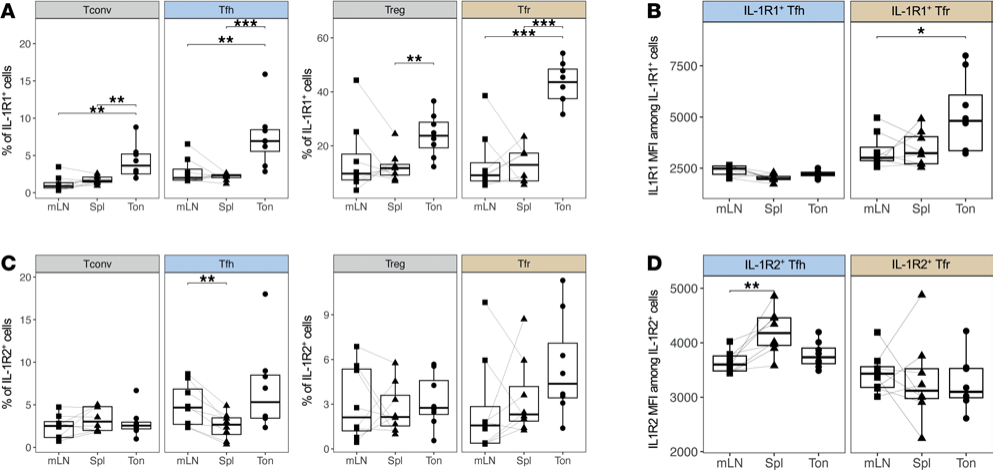
Tfh and Tfr display distinct patterns of IL-1Rs expression across secondary lymphoid organs. (**A**) Percentages of IL-1R1^+^ cells among CD4^+^ T cell subsets (*n* = 8 each). (**B**) Mean fluorescence intensity (MFI) of IL-1R1 within IL-1R1^+^ Tfh (left, blue) and Tfr (right, gold) across lymphoid organs (*n* = 8 each). (**C**) Percentages of IL-1R2^+^ cells among CD4^+^ T cell subsets (*n* = 8 each). (**D**) MFI of IL-1R2 within IL-1R2^+^ Tfh (left, blue) and Tfr (right, gold) across lymphoid organs (*n* = 8 each). In **A**–**D**, comparisons between mLN and Spleen have been assessed using a paired Wilcoxon test and comparisons between mLN and Spleen or Spleen and Tonsils have been assessed using a Mann-Whitney test. The FDR procedure has been applied for *P* value adjustments. *P* values are reported as the following: *P < 0.05; **P < 0.01; ***P < 0.001.

To investigate whether IL-1Rs contribute to the differential activation status of Tfol between lymphoid organs ex vivo, we first quantified the proportion of IL-1R1-expressing and IL-1R2-expressing cells among CD4^+^ T cells using flow cytometry (Supplemental Figure 2, A and B). Remarkably, the percentage of IL-1R1^+^ cells was significantly higher in tonsils, regardless of the CD4^+^ T cell subset, suggesting a link between increased tonsillar activation (Figure 1, C and D) and IL-1R1 expression (Figure 2A). Notably, IL-1R1 expression was preferentially observed in regulatory subsets, including Treg and Tfr, within all lymphoid organs (Supplemental Figure 2C). Similarly, the MFI of IL-1R1 among IL-1R1^+^ Tfh and Tfr is enhanced in tonsillar Tfr compared to mLN counterparts (*P* < 0.05) (Figure 2B).

The proportion of IL-1R2^+^ cells among CD4^+^ T cell subsets varied to a lesser extent between organs (Figure 2C). However, we did observe that IL-1R2^+^ Tfh from mLNs (4.7 ± 2.3%) were significantly more abundant than IL-1R2^+^ Tfh in the spleens (2.7 ± 1.1%) (*P* < 0.01). Thus, in Tfh, the expression of the IL-1 antagonist receptor matches that of proliferation and activation markers (Ki-67 and CD38) (Figure 1D). Of note, the proportion of cells expressing IL-1R2 was higher among Tfh than Tconv subsets in tonsils and mLNs (Supplemental Figure 2C). The MFI of IL-1R2 solely differed within IL-1R2^+^ Tfh, not Tfr counterparts, the former being more elevated within spleens than mLNs (P < 0.01) (Figure 2D). Interestingly, the proportion of IL-1R2^+^ Tfh appeared dissociated from the MFI, suggesting that a significant proportion of Tfh cells in mLNs express IL-1R2 weakly.

When comparing patterns of IL-1R1 and IL-1R2 expression between Tfh and Tfr across organs, Tfr cells, and not Tfh cells, seemingly displayed a multimodal expression of IL-1R1 with emphasis on tonsils (Supplemental Figure 2D). Such heterogeneity indicates that multiple Tfr subsets are found according to IL-1R1 expression and lymphoid activation.

Overall, the different IL-1R1 and IL-1R2 expression profiles between organs underline distinct phenotypes depending on the activation state of Tfh and Tfr cells.

### The expression of IL-1Rs captures a distinct, activated Tfr population and is associated with Tfh maturation.

We hypothesized that the expression of IL-1Rs could contribute to the different stages of follicular T cell activation. To assess this contribution, we merged ex vivo flow cytometry data from 8 tonsils and 8 paired spleens and mLNs to set up a metaorgan (*n* = 24) with the aim of comparing Tfh and Tfr activation stages according to their organ of origin. We focused our analysis on Tfr (CD4^+^ CXCR5^+^ Foxp3^+^), Tfh (CD4^+^ CXCR5^+^ Foxp3^–^), and Treg (CD4^+^ CXCR5^–^ Foxp3^+^), all 3 being the populations with higher IL-1R1 or IL-1R2 expression.

**Figure 3:**
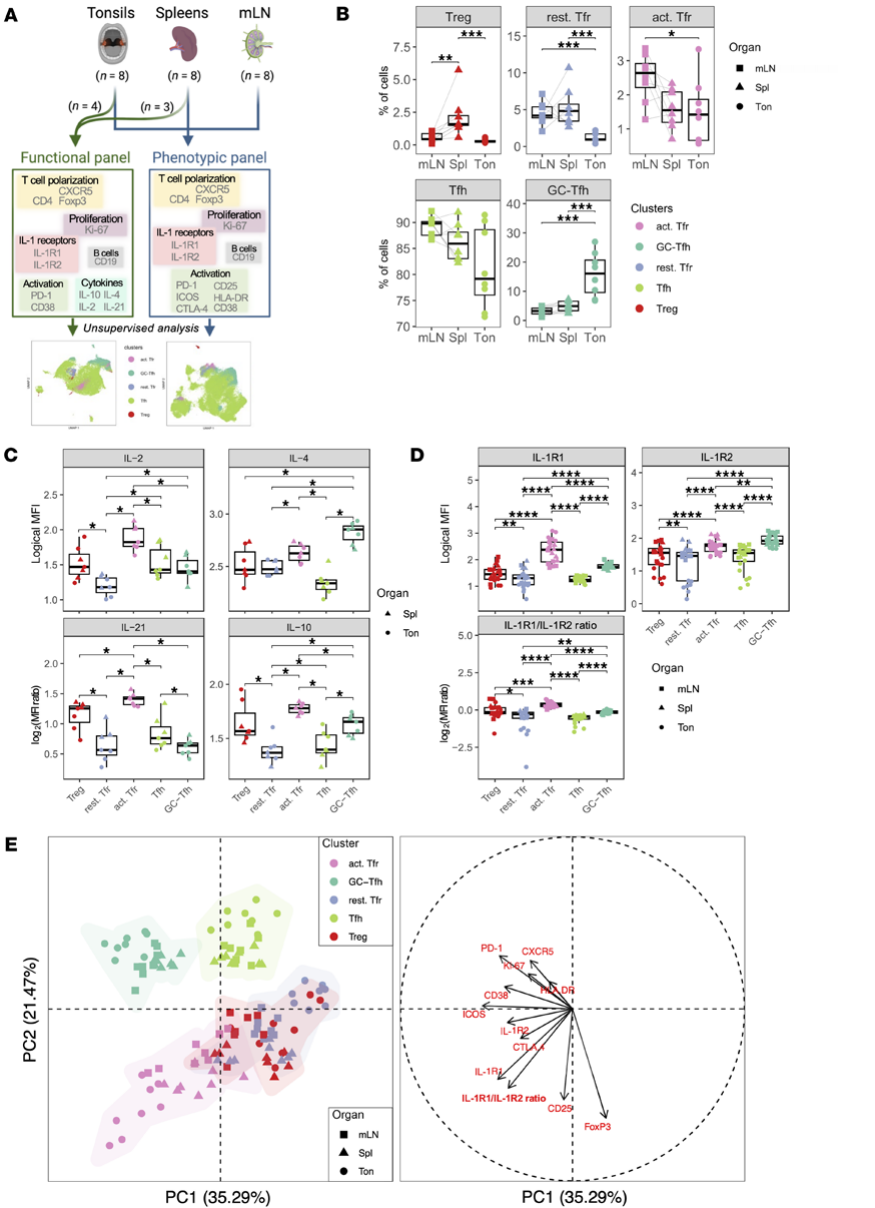
IL-1R1 and IL-1R2 differential expression defines an activated Tfr population and shapes Tfh maturation. (**A**) Experimental design of ex vivo lymphoid cell characterization using flow cytometry with a phenotypic panel (for 8 tonsils, 8 spleens, and 8 mLN) and a functional panel (for a selection of 4 tonsils and 3 spleens). (**B**) Frequency of each cluster among all CD4^+^ CXCR5^+^ and CXCR5^–^ Foxp3^+^ cells, across tonsils (Ton), Spleens (Spl), and mLN (*n* = 8 each). (**C**) Logicle-transformed MFI of IL-2, IL-4, IL-21, and IL-10 across the 5 clusters. (**D**) Logicle-transformed MFI of IL-1R1 and IL-1R2 (top), and ratio of IL-1Rs’ MFI (bottom) across the 5 clusters within all lymphoid organs (*n* = 24). (**E**) Principal component analysis (PCA) displaying the separation of samples according to the five clusters within all lymphoid organs (left) and its associated correlation circle showing the contribution of each marker to the principal components indicated with vectors (right). In **B**, comparisons between mLN and Spleen have been assessed using a paired Wilcoxon test,and comparisons between mLN and Spleen or Spleen and Tonsils have been assessed using a Mann-Whitney test. In **C** and **D**, comparisons have been assessed using Mann-Whitney test. The FDR procedure has been applied for *P* value adjustments. *P* values are reported as the following: *P < 0.05; **P < 0.01; ***P < 0.001; ****P < 0.0001.

To characterize the cells involved in our metaorgan, we used a phenotypic panel assessing activation markers and IL-1Rs across all samples (*n* = 24), alongside a functional panel measuring cytokine production on a selection of spleens (*n* = 3) and tonsils (*n* = 4) (Figure 3A). To enable an unbiased analysis of cell populations based on multiple markers, we applied unsupervised methods. Specifically, we performed UMAP dimensionality reduction using the ex vivo expression of 11 markers from the phenotypic panel (Supplemental Figure 3A) and 4 additional cytokines from the functional panel (Supplemental Figure 3B) to capture cellular heterogeneity within the metaorgan. Then, we identified clusters of interest based on an analytical strategy encompassing unsupervised clustering, metaclustering, and literature-based annotation (Supplemental Figure 3C). This allowed the identification of 5 clusters that discriminated cells upon their activation status: two Tfh clusters with increasing CXCR5 and PD-1 expression (namely Tfh and GC-Tfh), 2 Tfr clusters that differed in the expression of Ki-67, HLA-DR, ICOS, and CTLA-4 (namely resting Tfr and activated Tfr), and one Treg cluster (Supplemental Figure 3D). Indeed, activated Tfr (act. Tfr) phenotypically clustered together with GC-Tfh as a result of a shared expression of PD-1, Ki-67, HLA-DR, ICOS, and CD38, while the expressions of CD25, Foxp3, and CTLA-4 markers were rather shared with resting Tfr (rest. Tfr) and Treg clusters. Functionally, act. Tfr and Treg clustered together due to a similar pattern of Foxp3, Ki-67, IL-21, and IL-10 expression, as opposed to Tfh and rest. Tfr, which displayed a poor expression of these markers (Supplemental Figure 3E). The abundance of each cluster between SLOs revealed that the two Tfh clusters are not equally present, since GC-Tfh had higher frequencies in activated tonsils versus mLNs and spleens (*P* < 0.001) (Figure 3B). This confirms that tonsils display activated Tfol (Figure 1E) and attests to the relevance of the metaorgan clustering approach. In contrast, all regulatory cell clusters had significantly lower frequencies within tonsils (Figure 3B).

In detail, the quantification of cytokine expression revealed that act. Tfr produce the most amounts of cytokines (Figure 3C). In contrast, rest. Tfr and Tfh are the clusters with weaker cytokine production. Indeed, the act. Tfr cluster displayed higher expression of IL-2 (*P* < 0.05), IL-4 (*P* < 0.05), IL-21 (*P* < 0.05), and IL-10 (*P* < 0.05) than *rest*. *Tfr*, and higher expression of IL-2 (*P* < 0.05), IL-10 (*P* < 0.05), and IL-21 (*P* < 0.05) than GC-Tfh. The latter only showing increased expression of IL-4 relative to act. Tfr. These observations indicate that act. Tfr share GC effector functions with Tfh populations (IL-4 and IL-21), while also retaining suppressive properties in common with Treg (IL-10, IL-2). On the contrary, rest. Tfr lack IL-4 and IL-21 expression, suggesting a distinct phenotype.

Regarding the expression of IL-1Rs, IL-1R1 logicle-MFI was significantly higher in act. Tfr (*P* < 0.0001), with an even higher expression in tonsils (Figure 3D). *GC-Tfh* also displayed an increased IL-1R1 expression compared with *Tfh* (*P* < 0.0001). Accordingly, these activated clusters also upregulated IL-1R2 expression compared with their nonactivated counterparts (*P* < 0.0001). When performing the ratio of surface expression of IL-1R1/IL-1R2 MFIs, which assesses the responsiveness of cells to IL-1β, act. Tfr and GC-Tfh also displayed a significant increase compared with their resting counterparts (*P* < 0.0001). MFI ratios were consistent between and within SLOs for all clusters, except tonsil, which had higher variability due to a stronger activation status. Of note, solely act. Tfr had a positive median log_2_-transformed MFI ratio, thus indicating a more important sensitivity to IL-1β, while rest. Tfr are less likely to respond to IL-1β. Therefore, IL-1Rs appear as surrogate markers of Tfh and Tfr activation.

To assess the contribution of each marker to cluster distribution across organs, we performed a principal component analysis (PCA) on our 5 clusters (Figure 3E). The PCA separated Tfh from Tfr and Tregs well, though Tfr and Tregs were intermingled. PCA points towards a continuum from Tfh to GC-Tfh following CXCR5 and PD-1 expression, with tonsillar samples being the most driven by this activated phenotype compared with spleens and mLNs. Rest. Tfr strongly overlapped with the Treg cluster, indicating their phenotypical similarity. Strikingly, the act. Tfr cluster appeared more segregated with a marked distinction of tonsillar samples, due to the contribution of IL-1R1 and the IL-1R1/IL-1R2 ratio. Apart from the scattering of tonsillar act. Tfr, all other clusters and SLOs display high intraorgan consistency, therefore confirming the heterogeneity specifically associated with Tfr activation. Act.Tfr share markers with regulatory clusters (CTLA-4, CD25, Foxp3) and costimulation (ICOS). IL-1R2, on the other hand, partly drives the GC-Tfh cluster, suggesting that IL-1R2 expression on GC-Tfh contributes to the maintenance of a GC-like phenotype, while IL-1R1, rather, orientates to a Tfr phenotype. CD38 and ICOS, while contributing to the GC-Tfh cluster, are also shared with act. Tfr and appear as markers of follicular T cell activation.

Taken together, our data indicate that the Tfr population is heterogeneous, one subset being activated in accordance with the expression of IL-1R1 over IL-1R2. While Tfh activation is also associated with increased IL-1R1 expression, IL-1R2 seems critical for GC-Tfh maintenance by potentially modulating the sensitivity of the cells to IL-1β signaling. Act. Tfr cells are functionally close to both GC-Tfh subsets and Treg cells, indicating that IL-1β–responsive Tfr exhibit dual suppressive and effector functions.

### IL-1R1 and IL-1R2 expression is finely tuned during in vitro Tfol activation and IL-1β modulation.

**Figure 4:**
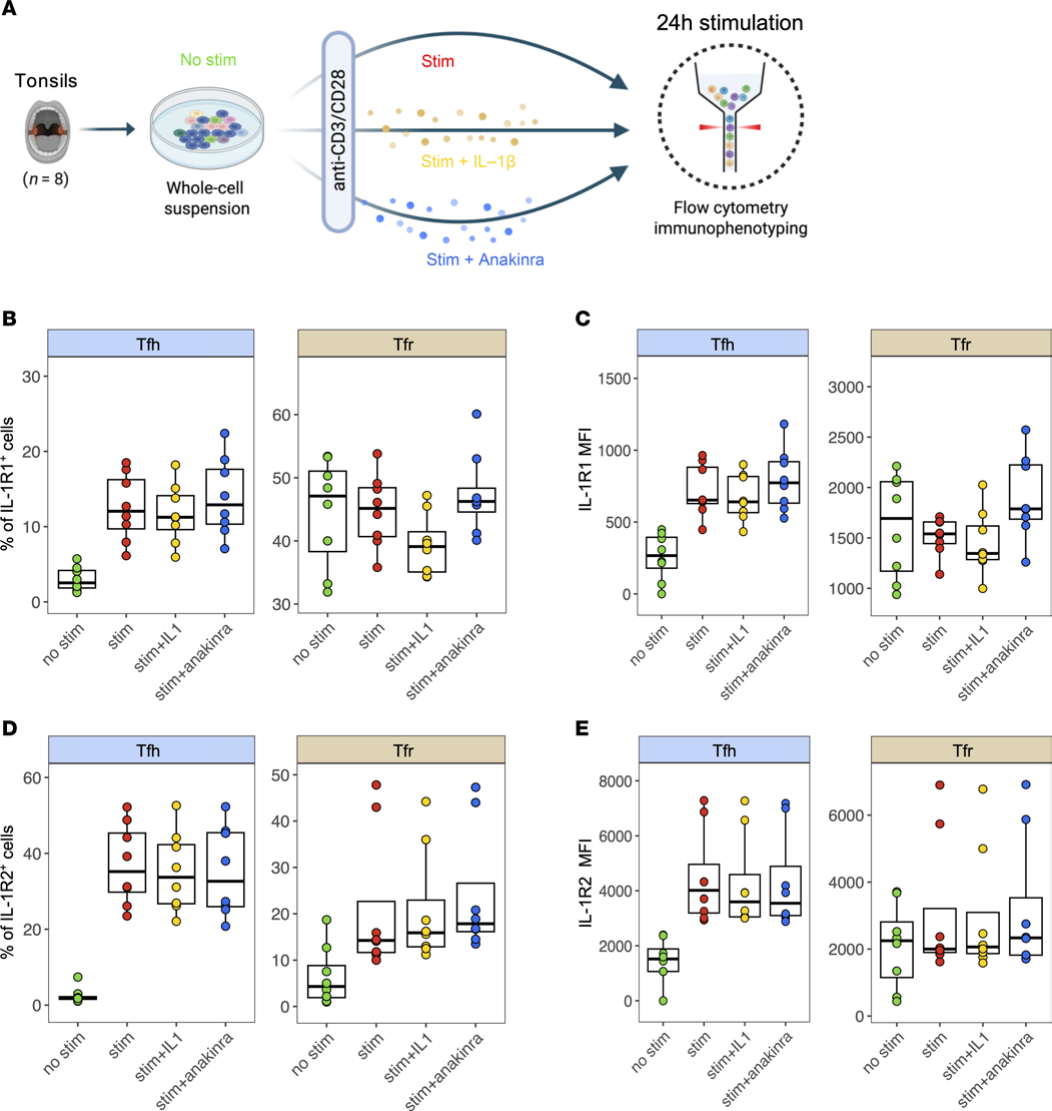
IL-1R1 and IL-1R2 expression in Tfh and Tfr is dynamically regulated by activation and IL-1β modulation. (**A**) Experimental design for whole-cell tonsillar suspensions (*n* = 8) stimulated with anti-CD3/CD28 beads for 24 hours, either in the presence of IL-1β (0.5 μg/mL), Anakinra (1 μg/mL), or alone. The expression of IL-1R1 and IL-1R2 was measured using flow cytometry. (**B**) Percentage of IL-1R1^+^ cells among Tfh (left) and Tfr (right) within each condition. (**C**) Boxplots showing the MFI of IL-1R1 among Tfh (left) and Tfr (right) from whole-cell tonsillar cultures compared to no stimulation (*n* = 8). (**D**) Percentage of IL-1R2^+^ cells among Tfh (left) and Tfr (right) within each condition. (**E**) Boxplots showing the MFI of IL-1R2 among Tfh (left) and Tfr (right) from whole-cell tonsillar cultures compared to no stimulation (*n* = 8). Comparisons have been assessed using Mann-Whitney test.

To investigate how Tfh and Tfr responsiveness to IL-1β is regulated during cell activation, we compared the expression of IL-1Rs between stimulated (stim) and unstimulated (no stim) Tfh and Tfr. We cultured total tonsillar lymphoid cells (*n* = 8) for 24 hours and stimulated T cells with anti-CD3/CD28 beads alone (stim) or with IL-1β (stim+IL-1β) or with IL-1R1 inhibitor Anakinra (stim+Anakinra) (Figure 4A).

First, only anti-CD3/28–stimulated Tfh displayed an increased percentage of IL-1R1^+^ cells compared to no stim (Figure 4B). IL-1R1^+^ Tfr did not exhibit variations upon stimulation alone, potentially due to their already high baseline expression (Figure 4C). However, adding IL-1β tended to decrease the percentage of IL-1R1^+^ Tfr compared to stim, while inhibiting endogenous IL-1β signaling with Anakinra tended to increase both IL-1R1^+^ Tfh and Tfr compared with IL-1β–treated conditions. This suggests that IL-1β is produced in vitro during stimulation and that Tfol adjust their IL-1R1 expression in response to IL-1β excess or deprivation (Figure 4C).

Similar to their IL-1R1 counterparts, IL-1R2^+^ Tfh were increased during stimulation alone but not IL-1R2^+^ Tfr (Figure 4D). Yet, none of the two populations exhibited variations of IL-1R2^+^ cells upon addition of IL-1β or Anakinra. Consistently, IL-1R2 expression levels mirrored these trends in both Tfh and Tfr, with stimulation alone driving IL-1R2 upregulation in Tfh, while neither IL-1β addition nor Anakinra treatment induced further changes (Figure 4E). Of note, the ratio of IL-1R1/IL-1R2 MFIs remained unchanged in all conditions among Tfh (Supplemental Figure 4A) and Tfr (Supplemental Figure 4B), indicating that sensitivity to IL-1β was maintained after stimulation. Similar trends of IL-1R1 and IL-1R2 expression were observed on isolated Tfh and Tfr cocultures stimulated with IL-1β, suggesting a direct effect (not shown). Regarding cell abundance, the Tfr/Tfh ratio did not significantly vary between conditions, although the addition of Anakinra tended to decrease the ratio, and thus did not favor Tfr (Supplemental Figure 4C).

In summary, IL-1R1 expression tended to be upregulated during the stimulation of Tfh. Following the addition of IL-1β, a negative feedback mechanism seemed to reduce IL-1R1 expression in both Tfh and Tfr. On the contrary, the addition of Anakinra tended to restore IL-1R1 expression without impacting IL-1R2 expression.

### IL-1β signaling shapes Tfh maturation and potentiates the transition to a Tfr phenotype.

**Figure 5:**
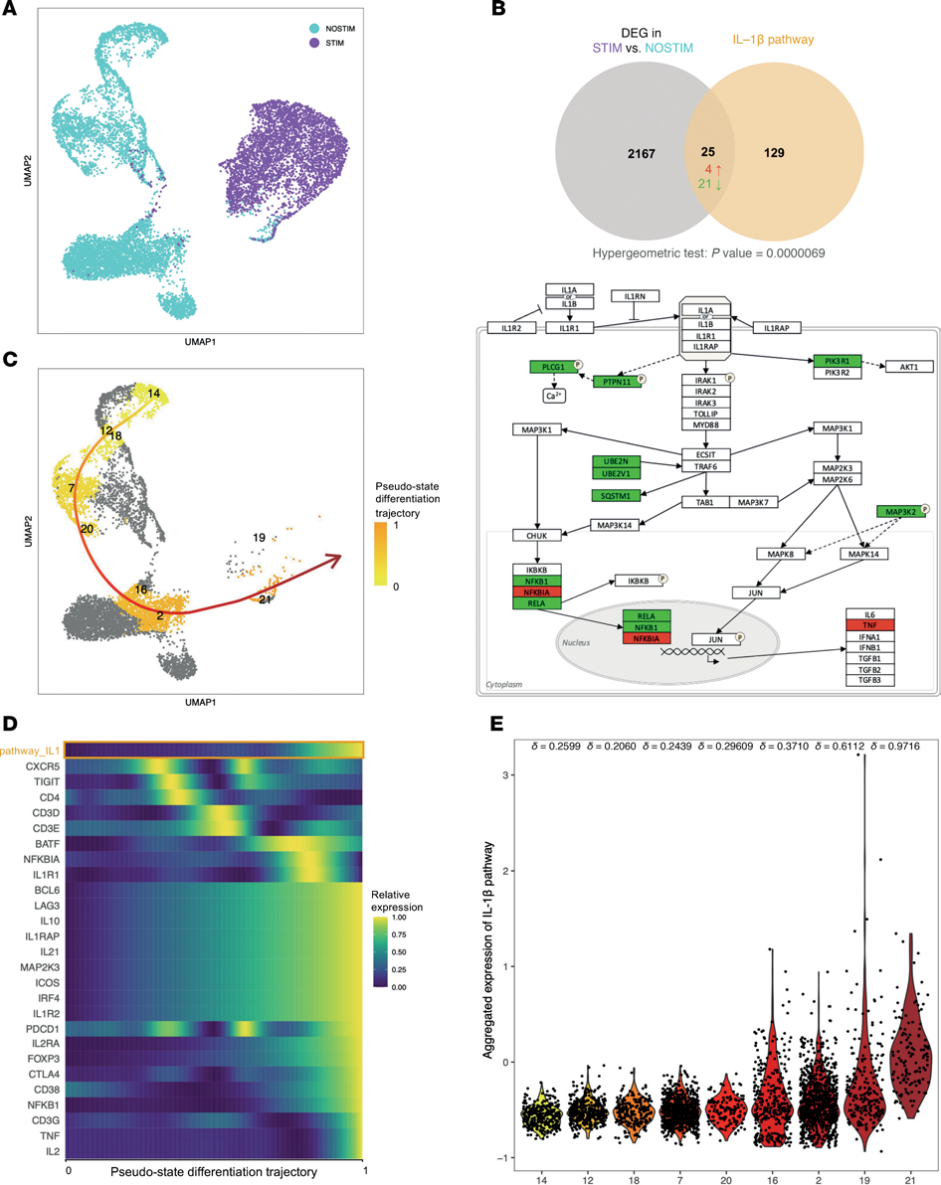
IL-1β signaling is associated with Tfh activation and transition to Tfr. (**A**) UMAP representation of CD3^+^CD4^+^ T cells from single-cell RNA-seq of whole-cell tonsillar suspensions (*n* = 2), either unstimulated (NOSTIM) or stimulated (STIM) with anti-CD3/CD28 beads for 24 hours. (**B**) Top: Venn diagram displaying the number of genes associated to the IL-1β pathway and found to be differentially expressed between STIM and NOSTIM conditions. The number of shared upregulated genes are indicated in red and the number of shared downregulated genes are represented in green. Bottom: Schematic representation of the downstream signalling of IL-1β pathway (WikiPathways WP4496 and WP195), annotated with the directionality of gene expression changes observed. (**C**) UMAP representation restricted to NOSTIM samples, showing an inferred pseudo-state trajectory from conventional T cells (Tconv, cluster #14) to activated follicular regulatory cells (act. Tfr, cluster #21). (**D**) Heatmap depicting the gradient of selected gene expression along the pseudo-state differentiation trajectory. (**E**) Violin plots illustrating aggregated IL-1β pathway expression across pseudo-state–ordered clusters involved in the inferred trajectory. d, Cliff’s delta effect size quantifying the magnitude of IL-1β pathway expression changes relative to cluster #14.

To confirm that follicular T cell activation is indeed associated with IL-1β signaling, we performed single-cell RNA sequencing (scRNAseq) on live tonsillar lymphoid cells (*n* = 2) in unstimulated (NOSTIM) and anti-CD3/CD28-stimulated (STIM) conditions for 24 hours. We selected helper T cells to perform dimensionality based on the expression of all genes, highlighting a clear segregation between NOSTIM and STIM samples (Figure 5A).

Then, we underwent gene expression analysis between unstimulated and stimulated conditions, revealing 2,167 differentially expressed genes (DEG), which we compared to 154 genes from the IL-1β pathway (Figure 5B). This exhaustive 154-gene list of the pathway was constituted by searching public databases (WikiPathways, Reactome, KEGG pathway) for all genes involved in sensing, processing, signaling, and regulation of IL-1β and its direct targets (Supplemental Table 1). The overlap between the DEG and genes belonging to the IL-1β pathway was statistically significant (*P* value < 0.0000069). Among the 154 genes composing the pathway, 25 were found among DEG, 4 of them being upregulated and 21 downregulated. To visualize the involvement of these genes within the pathway, we represented the downstream signaling of a curated public IL-1β pathway (WikiPathways WP4496) and annotated the directionality of gene expression (Figure 5B). Interestingly, key genes involved in signal transduction to the nucleus and further transcription (*NFKB1*, *NFKBIA*, *RELA*) belonged to the DEG, as well as *TNF*, a direct transcriptional target.

To further characterize the cell populations involved, we identified 23 distinct clusters of cells (Supplemental Figure 5A). These cell clusters were annotated based on the expression of key markers outlined in the cytometry annotation strategy (Supplemental Figure 3C), including *FOXP3*, *IL2RA* (CD25), *CXCR5*, *PDCD1* (PD-1), *ICOS*, *CTLA4*, *CD38*, *IL10*, *IL21*, and IL-1 receptor genes (Supplemental Figure 5B). As a result, we assigned 7 clusters to activated Tfr, 1 to resting Tfr, 5 to Tfh, 4 to GC-Tfh, and 6 to Tconv. Notably, no distinct Treg cluster was identified, likely due to their low frequency in tonsils (Figure 3B) and the limited input of cells in scRNA-seq compared with flow cytometry.

To investigate the relationships between follicular T cell clusters in tonsils, we performed a pseudo-state trajectory inference using cells belonging to the unstimulated condition. Defining cluster #14 (Tconv) as the starting point due to its low activation status, we identified a trajectory progressing through clusters #12 (Tconv), #18 (Tconv), #7 (Tfh), #20 (Tfh), #16 (GC-Tfh), and #2 (act. Tfr), ultimately leading to clusters #19 and #21 (act. Tfr) (Figure 5C). This trajectory suggests that a subset of GC-Tfh may transition into an activated Tfr phenotype, whereas rest. Tfr (cluster #10) are not encompassed in this differentiation pathway.

To determine the key genes involved in Tfh maturation and the transition to Tfr, we projected their relative expression along the pseudo-state trajectory (Figure 5D). The emergence of Tfh (cluster #7) is marked by an initial peak in *CXCR5* expression, followed by the sequential upregulation of *TIGIT*, *CD4*, *CD3*, and *BATF*. At later pseudo-states, *IL1R1* expression surges, preceding the upregulation of *BCL6*, *FOXP3*, *IL10*, *IL21*, *ICOS*, *CTLA4*, *CD38*, and *NFKB1*, which collectively define an activated Tfr phenotype characterized by both canonical transcription factors and a combination of suppressive and effector cytokines. At the final differentiation stage, *TNF* and *IL2* are upregulated — both known as direct targets of the IL-1β/NF-κB pathway. Notably, *IL1R1* upregulation precedes that of *IL1R2*, which gradually increases and peaks at later pseudo states. This pattern indicates that a high IL1R1/IL1R2 ratio is necessary to drive the transition from Tfh to activated Tfr.

To further assess IL-1β pathway dynamics, we quantified and aggregated the expression of the 154 associated genes within the clusters of the pseudo-state trajectory (Figure 5E). Clusters exhibited a progressive upregulation of the IL-1β pathway, with a moderate effect size starting from GC-Tfh (clusters #16 and #2, Cliff’s δ (CD) > 0.3) and a strong effect size starting from act. Tfr (clusters #19 and #21, CD > 0.6). These findings confirm that IL-1β signaling is progressively enhanced during Tfh maturation and peaks in GC-Tfh before contributing to the emergence of act. Tfr.

Together, our transcriptomic analysis highlights the role of IL-1β in supporting Tfh and Tfr activation via NF-κB signaling. The upregulation of *IL1R1* relative to *IL1R2* appears to be a prerequisite for Tfh-to-Tfr transition, driving the acquisition of both effector and suppressive functions.

### Increased peripheral IL-1β and cTfr as surrogate markers of autoantibody production in autoimmune patients.

Since IL-1β affects the activation and function of Tfol in vitro, we explored whether the production of different types of autoantibodies could be linked to disrupted Tfh and Tfr balance and elevated IL-1β levels. To address this question, we analyzed flow cytometry immunophenotyping, cytokine, and autoantibody detections from blood samples of a cohort of patients with 11 distinct autoimmune and autoinflammatory diseases, as well as healthy donors.

**Figure 6:**
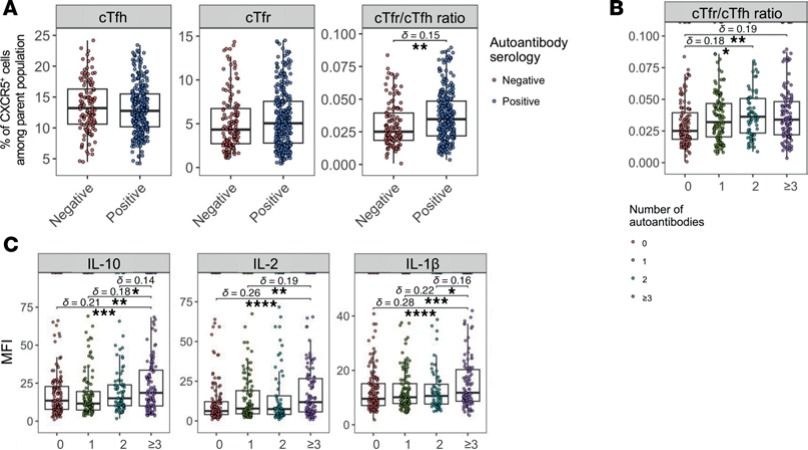
Higher autoantibody load is associated with increased cTfr and IL-1β–related molecules in the blood of autoimmune patients. (**A**) Frequency of circulating Tfh (cTfh) and circulating Tfr (cTfr) cells, as well as the cTfr/cTfh ratio, in relation to the autoantibody serology (Negative: *n* = 145; Positive: *n* = 324) of healthy volunteers and patients from the Transimmunom cohort. cTfh are CXCR5^+^ cells among CD3^+^ CD4^+^ T cells and cTfr are CXCR5^+^ cells among Foxp3^+^ CD127^–^ Treg. (**B**) cTfr/cTfh ratio among all individuals according to the number of autoantibody specificities (0: *n* = 145; 1: *n* = 122; 2: *n* = 75; ≥ 3: *n* = 127). (**C**) MFI measurements of peripheral cytokine levels according to the number of autoantibodies in all individuals. Comparisons have been assessed using Mann-Whitney test. The FDR procedure has been applied for *P* value adjustments. *P* values are reported as the following: *P < 0.05; **P < 0.01; ***P < 0.001; ****P < 0.0001. d, Cliff’s delta effect size. cTfr, circulating Tfr; cTfh, circulating Tfh; sIL-1R1, soluble IL-1R1; sIL-1R2, soluble IL-1R2.

We grouped individuals based on their autoantibody status and analyzed the levels of cTfr over cTfh cells (Figure 6A). While the percentages of cTfh and cTfr did not differ significantly between autoantibody-positive (*n* = 324) and -negative individuals (*n* = 145), the cTfr/cTfh ratio was higher in those who were autoantibody positive (Figure 6A). Among autoantibody-positive individuals, we quantified the number of different autoantibody specificities (from 1 to 8, 0, *n* = 145; 1, *n* = 123; 2, *n* = 75; ≥ 3, *n* = 127) and grouped those with 3 or more specificities (≥ 3) (Figure 6B). We found that higher autoantibody diversity was associated with an increased cTfr/cTfh ratio. Notably, we also found a significant correlation (*R* = 0.55 and *P* < 0.001) between cTfr and cTfh across all individuals (Supplemental Figure 6A).

We also measured the levels of IL-10, IL-2, and IL-1β and found that they were upregulated with higher autoantibody diversity (Figure 6C). Soluble IL-1R1 (sIL-1R1) levels were higher in individuals with more autoantibodies, while soluble IL-1R2 (sIL-1R2) levels remained unchanged (Supplemental Figure 6B). This suggests that cells undergoing strong activation and stimulation by IL-1β release IL-1R1 as a regulatory response to inflammation.

Overall, our findings indicate that higher autoantibody diversity is associated with increased peripheral IL-1β and cTfr levels and the release of sIL-1R1.

## Discussion

We describe the varying abundance and phenotypic heterogeneity of Tfol across 3 distinct lymphoid organs — tonsils, spleens, and mesenteric lymph nodes. Using ex vivo flow cytometry, we challenge the traditional static view of Tfh and Tfr by addressing their activation stages as a continuum, which is associated with increased expression of IL-1R1 at later stages. In Tfr cells, when IL-1R1 overcomes IL-1R2 expression, we describe an activated subset characterized by a shared phenotype and function with both Treg and GC-Tfh. scRNA-seq and in vitro stimulation underscore that follicular T cell activation requires IL-1β signaling through IL-1R1. As a result, Tfh and Tfr can vary the expression of IL-1R1 on their surface to modulate responsiveness to IL-1β. The exploration of IL-1β–related molecules on autoimmune individuals from the transimmunom cohort revealed that IL-1β, circulating Tfr and IL-10 are surrogate markers of higher autoantibody load.

In summary, we show that Tfr heterogeneity across lymphoid organs is linked to IL-1R1 and IL-1R2 differential expression. Follicular T cell activation through IL-1β signaling could orchestrate the emergence of a Tfr subset with dual suppressive and helper functions. Excess of IL-1β during GC reaction may promote Tfr emergence and autoreactive antibody production.

Our work demonstrates that GC-Tfh, which are classically thought to be an end stage of Tfh differentiation, appears as a potential seeder for the Tfr pool. As a result, 2 main Tfr subsets are found within human SLOs; one is activated, phenotypically close to GC-Tfh and characterized by the expression of IL-1R1, CD25, CTLA-4, CD38, and Ki-67, as well as the production of IL-10, IL-2, IL-21, and IL-4, while the other Tfr subset is present at a steady state and is phenotypically closer to Treg and Tfh. The phenotypic diversity observed among tonsillar Tfol may be influenced by the prior tonsillitis leading to surgery; however, this risk was minimized by exclusively collecting tonsils from patients without ongoing inflammatory conditions.

Although we did not experimentally assess trajectories leading to Tfr induction, a recent study demonstrates that GC-Tfh acquire CD25 and Foxp3 before differentiating into CD38^+^ CD25^+^ ICOS^+^ PD-1^+^ CTLA-4^+^ “iTfr,” located within the GC, clonally related to Tfh and able to promote B cell maturation via IL-10 production (22). These observations are in line with the phenotype of our act. Tfr cluster, which shares properties with both GC-Tfh and Treg. Our findings extend this mechanism and suggest that IL-1β could drive GC–Tfh-to-act. Tfr transition. In contrast, it was shown that “nTfr” (CD38^–^) were clonally related to Treg and retained suppressive functions while remaining at the T-B border, indicating that our resting Tfr could emerge from the Treg lineage independently of IL-1β signaling.

The existence of a Tfr subset with helper properties challenges the current dogma. However, there is increasing evidence of Tfr having a dual helper and suppressive role in the context of GC reaction (35–37). In particular, the role of CD25 has been debated over the past years, given the discordance between murine studies, stating that GC-resident Tfr lose CD25 expression (29, 38), while human studies assert the maintenance of CD25 expression (22, 39). Le Coz et al. demonstrated that transition from GC-Tfh to act. Tfr phenotype is enhanced by IL-2 supplementation in vitro (22). In line with those results in humans, we provide evidence that act. Tfr highly express CD25. We propose a model in which IL-1β also favors Tfr transition due to excess of IL-1R1 over IL-1R2 expression in Tfh, giving rise to a GC-resident, Tfh-derived Tfr population.

In our single-cell transcriptomics results, Tfol activation is associated with IL-1β/NF-κB signaling. Of note, when analyzing DEGs between unstimulated and stimulated conditions within T Fol only (excluding Tconv) most genes significantly enriched in the IL-1β pathway were upregulated (*P* < 0.0000011), contrasting with the present analysis, which includes Tconv (not shown and Figure 5B). Combined with aggregated expression of IL-1β pathway presented in Figure 5E, this observation confirms that during in vitro helper T cell activation, Tconv potentially downregulate IL-1β signaling while Tfol specifically upregulate it.

Monocytes, macrophages, dendritic cells, and B cells are known sources of IL-1β in human lymphoid organs (40–43). However, we were unable to identify IL-1β–producing cells in our scRNA-seq dataset. This limitation may be due to the low abundance of these cells in our lymphoid cell suspensions or the transient nature of IL-1β expression following proteolytic processing of its precursor rather than gene upregulation (44). Yet, in our in vitro tonsillar lymphoid cell stimulation model, inhibition with Anakinra resulted in increased IL-1R1 expression among Tfh, suggesting that IL-1β is endogenously produced by antigen-presenting cells.

In vitro stimulated tonsillar T cells manifested a removal of surface IL-1R1 when IL-1β is added, which may occur in case of excessive IL-1 agonism. This mechanism can be achieved through IL-1R1 internalization (45). Also, the receptor can be cleaved and secreted as a soluble form, thus sequestering extracellular IL-1β when present in high concentrations (46). On the contrary, culture with Anakinra tended to increase IL-1R1 surface expression.

Finally, we explored a cohort of individuals with distinct autoimmune and inflammatory conditions, as well as healthy volunteers, to investigate cTfh, cTfr, and IL-1β–related molecules. Circulating Tfol and GC-related cytokines serve as biomarkers of ongoing GC activity within tissues. In this study, we show that cTfh and cTfr amounts are correlated, with the cTfr/cTfh ratio increasing as autoantibody diversity rises. This increase in patients producing autoantibodies may reflect an ongoing and uncontrolled GC reaction, where elevated cTfr levels act as a feedback mechanism attempting to suppress inflammation.

Our experimental findings reveal distinct Tfr phenotypes with heterogeneous suppressive and helper properties. Under inflammatory conditions characterized by increased IL-1β, IL-10–producing Tfr may paradoxically promote rather than inhibit autoantibody production. Indeed, our data suggest that IL-1β supports cTfr differentiation in a way that sustains autoantibody production through IL-10, potentially contributing to autoimmune pathogenesis. These results challenge the dichotomic view of Tfr as purely protective against autoimmunity and Tfh as solely pathogenic by facilitating autoantibody production. Instead, cTfr may actively contribute to disease by promoting ectopic lymphoid structure formation at inflammation sites, thereby sustaining pathogenic GC reactions (23, 47, 48). Accordingly, we show that peripheral IL-10 levels increase with autoantibody diversity, suggesting that Tfr-derived IL-10 can promote B cell maturation and antibody production (49). Similarly, IL-1β levels followed the same pattern, indicating that cTfr expansion can be driven specifically by IL-1β–mediated differentiation of act. Tfr.

The increase of sIL-1R1 in the blood of individuals with a high number of autoantibodies is in line with decreased surface IL-1R1 expression upon culture with IL-1β. The release of sIL-1R1 in the blood of individuals could be linked to excessive IL-1β binding and thus shedding of the receptor, serving as a biomarker of inflammation and autoantibody production. In contrast, decreased sIL-1R2 represents a lack of suppressive activity, which is correlated with joint destruction in arthritis (50). Inflammation-induced IL-1β could promote the differentiation of a specific act. Tfr subset endowed with GC-Tfh functions.

Overall, our unique approach combining the phenotype of distinct lymphoid organs with different activation states has enabled us to characterize follicular T cell heterogeneity involving IL-1β-responsiveness, which appears to be a central cytokine for Tfh and Tfr modulation during human GC reaction.

## Methods

### Sex as a biological variable.

Healthy organ donors, as well as patients and healthy volunteers from the Transimmunom observational trial, included both females and males. No marked differences were reported in our findings between the 2 sexes.

### Collection of human secondary lymphoid organ samples.

Tonsils were collected from 8 immunocompetent children undergoing partial tonsillectomy as a treatment for obstructive sleep apnea syndrome at Necker-Enfants Malades Hospital, Paris, France. These individuals were considered healthy, as no signs of infection or inflammation were present at the time of surgery. We analyzed 8 tonsils from children with a mean age of 4.67 ± 1.63 years, ranging in age from 3 to 7 years, and including 4 male and 4 female donors. Paired mesenteric lymph nodes and spleens were obtained from 8 adult organ donors undergoing liver removal for transplantation in digestive surgery department at Pitié-Salpêtrière Hospital, Paris, France. These individuals were considered healthy despite brain death, as they met the stringent criteria for organ donation. This included the absence of infection, malignancy, or conditions that might impair immune homeostasis. The average age of the organ donors was 56.8 ± 23.58 years, ranging from 21 to 92 years, with 4 male and 4 female donors.

### Dissociation of secondary lymphoid organs and lymphoid cell isolation.

Mesenteric lymph nodes (*n* = 8), spleens (*n* = 8), and tonsils (*n* = 8) were all treated following the same cell dissociation protocol. First, freshly collected samples were briefly washed with ethanol, sliced into pieces, and then transferred into C tubes (Miltenyi) containing complete medium: RPMI-1640 medium (Sigma-Aldrich) supplemented with 10% FBS, antibiotics (100 U Penicillin and 100 μg Streptomycin), and L-Glutamine. Cell dissociation was performed using a gentleMACS dissociator (Miltenyi), and the tube contents were filtered through 70 μm cell strainers. Mononuclear cells were isolated using a density gradient (Histopaque-1077, Merck). Cell viability was assessed using trypan blue staining. The cell suspensions were frozen in cryotubes with FBS and 10% DMSO and stored at –80°C for short-term experiments and in liquid nitrogen for long-term preservation.

### Phenotypic profiling of human secondary lymphoid organs.

Cell suspensions from mesenteric lymph nodes (*n* = 8), spleens (*n* = 8), and tonsils (*n* = 8) were thawed and left to rest overnight in a complete medium. The cells were then stained for viability using 405/420 Viobility (1/500, Miltenyi), washed, and stained with the following anti-human antibodies: CD3 (PerCP-Vio700, 1/100, Miltenyi, Cat# 130-109-465), ICOS (Vioblue, 1/100, Miltenyi, Cat# 130-100-737), CXCR5 (PE-Vio770, 1/500, Miltenyi, Cat# 130-117-358), CD4 (APC-Vio770, 1/500, Miltenyi, Cat# 130-113-223), CD38 (BV711, 1/400, BioLegend, Cat# 303527), CD25 (APC, 1/160, BioLegend, Cat# 302610), HLA-DR (BV785, 1/20, BD Biosciences, Cat# 564041), CD19 (PE-CF594, 1/200, BD Biosciences, Cat# 562294), IL-1R1 (PE, 1/80, R&D Systems, Cat# FAB269P), IL-1R2 (AF700, 1/80, R&D Systems, Cat# FAB663N), and PD-1 (PE-Cy5.5, 1/320, Beckman Coulter, Cat# B36123). Staining was performed in 1× PBS with 5% FBS and incubated in the dark for 30 minutes. The cells were fixed using the BD Pharmingen Transcription Factor kit according to the manufacturer’s protocol. Intracellular staining was performed after fixation and permeabilization (eBioscience Foxp3 Transcription factor staining buffer set, Invitrogen), according to manufacturer’s instructions, with the following anti-human antibodies: Ki67 (BUV395, 1/160, BD Biosciences, Cat# 564071), Foxp3 (AF488, 1/20, BD Biosciences, Cat# 560047), and CTLA4 (BV605, 1/40, BioLegend, Cat# 369610). The cells were incubated for 40 minutes at 4°C, washed with the washing buffer, resuspended in 1× PBS, and analyzed using a CytoFLEX LX cytometer (Beckman Coulter).

### Functional profiling of human secondary lymphoid organs.

Cell suspensions from selected spleens (*n* = 3) and tonsils (*n* = 4) were thawed and left to rest overnight in a complete medium at 37°C and 5% CO_2_. To enhance cytokine detectability, the cells were treated with phorbol 12-myristate 13-acetate (PMA) (1 μg/mL, Sigma-Aldrich) and ionomycin (iono) (1 μg/mL, Sigma-Aldrich) for 1 hour at 37°C and 5% CO_2_, and brefeldin A (10 μg/mL, Sigma-Aldrich) was added for 5 hours. Then, the cells were washed twice and stained with the following antibodies: CXCR5 (PE-Vio770, 1/500, Miltenyi, Cat# 130-117-358) and CD4 (APC-Vio770, 1/500, Miltenyi, Cat# 130-113-223). The cells were then fixed using the BD Pharmingen Transcription Factor kit. Intracellular staining was performed after fixation and permeabilization (eBioscience Foxp3 Transcription factor staining buffer set, Invitrogen), according to manufacturer’s instructions, with the following anti-human antibodies: Ki67 (BUV395, 1/160, BD Biosciences, Cat# 564071), Foxp3 (AF488, 1/20, BD Biosciences, Cat# 560047), CD38 (BV711, 1/400, BioLegend, Cat# 303527), CD19 (PE-CF594, 1/200, BD Biosciences, Cat# 562294), IL-1R1 (PE, 1/80, R&D Systems, Cat# FAB269P), IL-1R2 (AF700, 1/80, R&D Systems, Cat# FAB663N), PD1 (PE-Cy5.5, 1/320, Beckman Coulter, Cat# B36123), IL-4 (PerCP-Cy5.5, 1/160, BD Biosciences, Cat# 561234), IL-21 (APC, 1/200, Miltneyi, Cat# 130-117-422), IL-10 (BV421, 1/160, Biolegend, Cat# 501422), and IL-2 (BV605, 1/160, Biolegend, Cat# 500332). The cells were incubated for 40 minutes at 4°C, washed with the washing buffer, resuspended in 1× PBS, and analyzed using a CytoFLEX LX cytometer (Beckman Coulter).

### In vitro activation of tonsillar whole cell suspensions.

Frozen cell suspensions were thawed in complete medium, washed twice, and live cells were counted using trypan blue staining. The cells were then incubated at 37°C and 5% CO_2_ overnight prior to cell activation. Whole-cell suspensions were centrifuged and resuspended in a complete medium with anti-CD3/CD28 activation beads (Dynabeads Human T-Activator CD3/CD28, Thermo Fisher Scientific) at a concentration of 1 μL per 80,000 cells. Suspensions from each sample were then distributed into a 48-well plate with 500,000 cells per well. Three tonsillar conditions for cell activation were established: (a) stimulation alone, (b) stimulation with IL-1β premium grade (0.5 μg/mL, Miltenyi), and (c) stimulation with anti-IL-1 (Anakinra) (1 μg/mL, Kineret, SOBI). The plates were incubated at 37°C and 5% CO_2_ for 24 hours. After the incubation, cells were resuspended in 1× PBS prior to flow cytometry staining.

### Phenotypic profiling of cultured tonsillar whole cell suspensions.

Both unstimulated cells (as controls) and cultured whole-cell suspensions were then stained for viability using 405/420 Viobility (1/500, Miltenyi), washed, and stained with the following anti-human antibodies: ICOS (Vioblue, 1/100, Miltenyi, Cat# 130-100-737), CD3 (PerCP-Vio700, 1/100, Miltenyi, Cat# 130-109-465), CXCR5 (PE-Vio770, 1/500, Miltenyi, Cat# 130-117-358), CD19 (APC, 1/500, Miltenyi, Cat# 130-110-250), CD4 (APC-Vio770, 1/500, Miltenyi, Cat# 130-113-223), HLA-DR (BV785, 1/20, BD Biosciences, Cat# 564041), IL-1R1 (PE, 1/80, R&D Systems, Cat# FAB269P), IL-1R2 (AF700, 1/80, R&D Systems, Cat# FAB663N), CTLA-4 (BV605, 1/40, BioLegend, Cat# 369610), and PD-1 (PE-Cy5.5, 1/320, Beckman Coulter, Cat# B36123). Staining was performed in 1× PBS with 5% FBS and incubated in the dark for 30 minutes. The cells were then washed and fixed and permeabilized (eBioscience Foxp3 Transcription factor staining buffer set, Invitrogen), according to manufacturer’s instructions, and incubated for 45 minutes. Subsequently, the cells were washed with the kit washing buffer and stained with the intracellular mix containing the following anti-human antibodies: Ki67 (BUV395, 1/160, BD Biosciences, Cat# 564071) and Foxp3 (AF488, 1/20, BD Biosciences, Cat# 560047). The cells were incubated for 40 minutes at 4°C, washed again with the washing buffer, resuspended in 1× PBS, and analyzed using a Cytoflex LX cytometer (Beckman Coulter).

### Supervised analysis of flow cytometry data.

Raw cytometry data were compensated using CytExpert software (Beckman Coulter). Manual gating was performed using FlowJo software (BD), from which geometric mean fluorescence intensity (MFI) and percentages relative to parent populations were extracted for each population of interest. Additional gates were created to determine marker positivity and extracted for each relevant cell population.

### Unsupervised analysis of flow cytometry data.

Unsupervised analyses were performed on different cell populations, including CD4^+^ CXCR5^+^ Tfol, CD4^+^ Foxp3^+^ regulatory cells, and combinations of both. All subsequent analyses were conducted using R (version 4.3). Fluorescence intensity of all cells was transformed using the logicle method, and markers used solely for gating (Viability, CD3, CD19, CD4) were discarded. UMAP dimensionality reduction was performed using the uwot R package (https://cran.r-project.org/web/packages/uwot). Clustering was based on marker fluorescence intensity using the k-means algorithm, and resulting clusters were refined to reduce the final number via hierarchical-based metaclustering and supervised annotation. Heatmaps were generated with median expression values using the ComplexHeatmap R package (https://bioconductor.org/packages/release/bioc/html/ComplexHeatmap.html). Multidimensional scaling (MDS) and principal component analysis (PCA) were computed based on marker median expression values using the MASS R package (https://cran.r-project.org/web/packages/MASS) and the stats R package (https://stat.ethz.ch/R-manual/R-devel/library/stats).

### Sorting of live lymphocytes from cultured tonsillar whole cell suspensions.

Following 24-hour in vitro incubation of tonsillar cell suspensions under stimulation alone or left untreated, the cells were stained for viability using 405/520 Viobility (1/500, Miltenyi). Subsequently, the cells were washed with 1× PBS and 5% FBS. Sorting of live lymphocytes (LiveDead^–^ FSC-A^lo^ SSC-A^lo^) was performed using a BD FACSAria II sorter (BD Biosciences).

### Single-cell RNA library construction and sequencing.

Single-cell RNA library construction and sequencing were performed following the sorting of total live lymphocytes by flow cytometry: a single-cell library construction was performed using the Chromium Next GEM Single Cell 5′ Library and Gel Bead Kit v1.1 (PN-1000165), Chromium Next GEM Chip G (PN-1000127), Chromium Single-Cell 5′ Library Construction Kit (16 reactions) (PN-1000020), and Single Index Kit T Set A (96 reactions) (PN-1000123), according to the manufacturer’s protocol. Single-cell suspensions obtained from 20,000 cells, with barcoded gel beads and partitioning oil, were loaded onto Chromium Next GEM Chip G to generate single-cell bead-in emulsion. Full-length cDNA along with cell barcode identifiers were then PCR amplified to generate 5′ Gene Expression (GEX) libraries. These libraries were sequenced using a NovaSeq 6000 (Illumina) to achieve a minimum of 23,000 paired-end reads per cell for GEX. Reads were subsequently aligned to the GRCh38 human reference genome using Cell Ranger (v6.1.1, https://support.10xgenomics.com/single-cell-gene-expression/software/pipelines/latest/installation).

### Analysis of single-cell RNA-seq.

The expression matrices resulting from reads alignment were analyzed using the Seurat R package (51). Only high-quality cells were selected for subsequent analysis. The criteria for high-quality cells included a mitochondrial gene percentage below 10% and a number of unique molecular identifiers (UMI) between 200 and 15,000. Relative counts were used for data normalization. Dimensionality reduction was performed using principal component analysis (PCA) based on the 5,000 most variable genes. Helper T cells were isolated by selecting the cells matching the following rules for the number of reads: *CD3E* > 0 or *CD3D* > 0 or *CD3G* > 0 and *CD19* = 0 and *MS4A1* = 0 and *CD8A* = 0 and *CD8B* = 0. Differential gene expression analysis was conducted using a logistic regression model with *P* values corrected using the false discovery rate (FDR) procedure. Clustering based on a shared nearest neighbor (SNN) modularity, with a resolution parameter equals to 1.5, allowed the identification of 23 clusters (#0 to #22). Pseudo-state trajectory inference was performed on unstimulated samples using the Slingshot algorithm (52) with #14 (Tconv) as starting cluster. We used the Cliff’s Delta to quantify the magnitude of changes in IL-1β–pathways gene expression across different cell clusters relative to cluster #14. Cliff’s Delta is a nonparametric effect size measure that quantifies the degree of shift between two distributions, where values close to ± 1 indicate a strong difference, and values near 0 suggest minimal change.

### Transimmunom study design and cohort description.

Autoimmune and autoinflammatory patients as well as healthy volunteers were enrolled in the Transimmunom observational trial (NCT02466217) (53). In our study, we analyzed 469 participants, each from a condition accounting for at least 8 individuals: rheumatoid arthritis (*n* = 89); type 1 diabetes mellitus (*n* = 59); ankylosing spondylitis (*n* = 55); osteoarthritis (*n* = 47); Behçet disease (*n* = 38); systemic lupus erythematosus (*n* = 31); type 2 diabetes mellitus (*n* = 30); antiphospholipid syndrome (*n* = 23); Takayasu arteritis (*n* = 22); granulomatosis with polyangiitis (*n* = 8); Crohn’s disease (*n* = 10); and healthy volunteers (*n* = 57). Exclusion criteria for healthy volunteers were based on medical records, including cancer, autoimmune disease, acute or chronic infection, current pregnancy or any contraindication to blood donation. There were 273 females (58.2%) and 196 males (41.8%), with a mean age of 44.9 years (SD = 15.2), a mean body mass index of 25.7 kg/m^2^ (SD = 5.3), and a mean disease duration of 56.9 months (SD = 67.1). The most frequent secondary diagnoses included Sjogren’s syndrome (*n* = 19), osteoarthritis (*n* = 19), type 2 diabetes mellitus (*n* = 13), and ankylosing spondylitis (*n* = 11). Concomitant treatments were found in 303 participants (64.6%) with a highly heterogeneous distribution, showing no consistent pattern between and within conditions, and no substantial impact on the clinicobiological variables studied. The most frequent treatments were traditional disease-modifying antirheumatic drugs (*n* = 129; 27.5%); corticosteroids (*n* = 100; 21.3%); immunosuppressants (*n* = 97; 20.7%); cardiovascular treatments (*n* = 82; 17.5%); analgesics (*n* = 81; 17.3%); proton pump inhibitors (*n* = 66; 14.1%); antidiabetic insulin (*n* = 66; 14.1%); antirachitic vitamin D (*n* = 63; 13.4%); and nonsteroidal antiinflammatory drugs (*n* = 59; 12.6%).

### Blood sample management in the Transimmunom study for autoantibody, cytokine and cytometry assays.

Blood samples were collected from all participants to undergo multiple assays, performed by the Clinical Investigation Center for Biotherapies. For autoantibody quantification, performed in a hospital routine laboratory, 5 mL of serum was drawn into dry tubes. Multiplex enzyme-linked immunosorbent assay (ELISA) was used to detect 45 autoantibodies (Supplemental Table 2). The positivity threshold for each autoantibody was determined according to the standard reference values established by the laboratory conducting the analysis. Detected autoantibodies included antinuclear antibodies (anti-SSA52, anti-SSA60, anti-SSB, anti-Sm, anti-RNP, anti-Ro52, anti-nuclear DNA, anti-PmScl), anti-Ku, anti-TIF1, anti-cardiolipin IgG and IgM, anti-B2GP1 IgG and IgM, anti-Saccharomyces cerevisiae IgG and IgA, ANCA, anti-MPO, anti-PR3, anti-CCP, Latex, Waaler Rose, and anti-MBG. Cytokines and soluble receptors related to Tfh and Tfr activation, including IL-1β, IL-10, IL-2, sIL-1R1, and sIL-1R2, were quantified in the sera using multiplex assays (MILLIPLEX MAP Human Cytokine/Chemokine Growth Factor Panel A and Human Soluble Cytokine Receptor kits) with a MAGPIX analyzer according to the manufacturers’ instructions. Flow cytometry, performed with the Duraclone technology (Beckman Coulter) as described in Pitoiset et al. (54), enabled the identification and quantification of cell populations (55, 56), among which only Tfh and Tfr were considered.

### Statistics.

After normality testing by Shapiro-Wilk test, *P* values were calculated with Wilcoxon test for paired data and Mann-Whitney test for unpaired data. When 3 or more comparisons were made, adjusted *P* values were calculated using the false discovery rate (FDR) method. The statistical overrepresentation of gene list overlap with specific signatures was estimated using the hypergeometric test. Statistics provided in the results section are presented as median ± (1.58×IQR)/√n, where IQR represents the interquartile range and *n* the sample size.

### Study approval.

The collection and use of pediatric tonsils were authorized by the ethics committee of Sorbonne University (CER-2021-043). The collection and use of samples from adult organ donors were authorized under protocol PFS-14-009 by the French Biomedicine Agency. The Transimmunom observational trial was approved ethics committee Ile-De-France 48-15. Written informed consent was obtained from all participants in accordance with the Declaration of Helsinki and good clinical practice.

### Data availability.

Raw cytometry and Luminex data are available from the corresponding author upon request. Raw scRNA-seq data are available on the NCBI Sequence Read Archive (SRA) database under Bioproject accession number PRJNA1245656. R code used in the study is available upon request. Values for all data points in graphs are reported in the Supporting Data Values file.

## Author contributions

BB, DK, NT, PE, RJM, RV, and SGD conceptualized the study. NT, RJM, RV, and SGD designed the study methodology. AB, PE, GF, BB, and DK discussed study design and interpretation of data. BG, LP, NC, RJM, RV, and SAM performed the experiments. HD, NT, RV, and SGD analyzed the data. HV, MB, and JD provided bioinformatics support. NT, RV, and SGD wrote the manuscript. CR, FD, MR, R Luscan, and R Lorenzan recruited and enrolled patients. DK and SGD aquired funding. NT and SGD contributed equally as senior authors to this work. All authors reviewed, edited and approved the manuscript.

## Supplementary Material

Supplemental data

Supporting data values

## Version 1 05/20/2025

In-Press Preview

## Version 2 06/23/2025

Electronic publication

## References

[1] Young C, Brink R (2021). The unique biology of germinal center B cells. Immunity.

[2] Berek C (1991). Maturation of the immune response in germinal centers. Cell.

[3] Jacob J (1991). Intraclonal generation of antibody mutants in germinal centres. Nature.

[4] Nurieva RI (2009). Bcl6 mediates the development of T follicular helper cells. Science.

[5] Johnston RJ (2009). Bcl6 and Blimp-1 are reciprocal and antagonistic regulators of T follicular helper cell differentiation. Science.

[6] Yu D (2009). The transcriptional repressor Bcl-6 directs T follicular helper cell lineage commitment. Immunity.

[7] Crotty S (2019). T follicular helper cell biology: a decade of discovery and diseases. Immunity.

[8] Zhang Y (2013). Germinal center B cells govern their own fate via antibody feedback. J Exp Med.

[9] Wing JB (2018). Control of germinal center responses by T-follicular regulatory cells. Front Immunol.

[10] Maceiras AR (2017). T follicular helper and T follicular regulatory cells have different TCR specificity. Nat Commun.

[11] Linterman MA (2011). Foxp3^+^ follicular regulatory T cells control the germinal center response. Nat Med.

[12] Wollenberg I (2011). Regulation of the germinal center reaction by Foxp3^+^ follicular regulatory T cells. J Immunol.

[13] Chung Y (2011). Follicular regulatory T cells expressing Foxp3 and Bcl-6 suppress germinal center reactions. Nat Med.

[14] Dhaeze T (2015). Circulating follicular regulatory T cells are defective in multiple sclerosis. J Immunol.

[15] Cao G (2020). An imbalance between blood CD4^+^CXCR5^+^Foxp3^+^ Tfr cells and CD4^+^CXCR5^+^Tfh cells may contribute to the immunopathogenesis of rheumatoid arthritis. Mol Immunol.

[16] Jacquemin C (2015). OX40 ligand contributes to human lupus pathogenesis by promoting T follicular helper response. Immunity.

[17] Rubtsov YP (2008). Regulatory T cell-derived interleukin-10 limits inflammation at environmental interfaces. Immunity.

[18] Xie MM (2020). T follicular regulatory cells and IL-10 promote food antigen-specific IgE. J Clin Invest.

[19] Laidlaw BJ (2017). Interleukin-10 from CD4^+^ follicular regulatory T cells promotes the germinal center response. Sci Immunol.

[20] Jacobsen JT (2021). Expression of Foxp3 by T follicular helper cells in end-stage germinal centers. Science.

[21] Aloulou M (2016). Follicular regulatory T cells can be specific for the immunizing antigen and derive from naive T cells. Nat Commun.

[22] Le Coz C (2023). Human T follicular helper clones seed the germinal center-resident regulatory pool. Sci Immunol.

[23] Havenar-Daughton C (2016). CXCL13 is a plasma biomarker of germinal center activity. Proc Natl Acad Sci U S A.

[24] Almanan M (2020). IL-10-producing Tfh cells accumulate with age and link inflammation with age-related immune suppression. Sci Adv.

[25] Eto D (2011). IL-21 and IL-6 are critical for different aspects of B cell immunity and redundantly induce optimal follicular helper CD4 T cell (Tfh) differentiation. PLoS One.

[26] Dinarello CA (2018). Overview of the IL-1 family in innate inflammation and acquired immunity. Immunol Rev.

[27] Nakae S (2001). Interleukin-1 beta, but not interleukin-1 alpha, is required for T cell-dependent antibody production. Immunology.

[28] Ben-Sasson SZ (2009). IL-1 acts directly on CD4 T cells to enhance their antigen-driven expansion and differentiation. Proc Natl Acad Sci U S A.

[29] Ritvo P-GG (2017). Tfr cells lack IL-2Rα but express decoy IL-1R2 and IL-1Ra and suppress the IL-1–dependent activation of Tfh cells. Sci Immunol.

[30] Dolence JJ (2018). Airway exposure initiates peanut allergy by involving the IL-1 pathway and T follicular helper cells in mice. J Allergy Clin Immunol.

[31] Barbet G (2018). Sensing microbial viability through bacterial RNA augments T follicular helper cell and antibody responses. Immunity.

[32] Belbezier A (2024). Interleukin-1 regulates follicular T cells during the germinal center reaction. Front Immunol.

[33] Engeroff P (2024). IL-1R2 expression in Tfr cells controls allergic anaphylaxis by regulating IgG versus IgE responses. Allergy.

[34] Kothari H (2021). Identification of human immune cell subtypes most responsive to IL-1β-induced inflammatory signaling using mass cytometry. Sci Signal.

[35] Panneton V (2023). ICOS costimulation is indispensable for the differentiation of T follicular regulatory cells. Life Sci Alliance.

[36] Fonseca VR (2017). Human blood T_fr_ cells are indicators of ongoing humoral activity not fully licensed with suppressive function. Sci Immunol.

[37] Graca L (2023). The expanding family of T follicular regulatory cells. Sci Immunol.

[38] Wing JB (2017). A distinct subpopulation of CD25^–^ T-follicular regulatory cells localizes in the germinal centers. Proc Natl Acad Sci U S A.

[39] Sayin I (2018). Spatial distribution and function of T follicular regulatory cells in human lymph nodes. J Exp Med.

[40] Hadadi E (2016). Differential IL-1β secretion by monocyte subsets is regulated by Hsp27 through modulating mRNA stability. Sci Rep.

[41] Li S (2022). IL-1β expression in bone marrow dendritic cells is induced by TLR2 agonists and regulates HSC function. Blood.

[42] https://www.proteinatlas.org/ENSG00000125538-IL1B/single+cell/lymph+node.

[43] Bertoglio JH (1988). B-cell-derived human interleukin 1. Crit Rev Immunol.

[44] Afonina IS (2015). Proteolytic processing of interleukin-1 family cytokines: variations on a common theme. Immunity.

[45] Bourke E (2003). IL-1 beta scavenging by the type II IL-1 decoy receptor in human neutrophils. J Immunol.

[46] Elzinga BM (2009). Interleukin-1 receptor type 1 is a substrate for gamma-secretase-dependent regulated intramembrane proteolysis. J Biol Chem.

[47] Fonseca VR (2018). The ratio of blood T follicular regulatory cells to T follicular helper cells marks ectopic lymphoid structure formation while activated follicular helper T cells indicate disease activity in primary Sjögren’s syndrome. Arthritis Rheumatol.

[48] Sage PT (2014). Circulating T follicular regulatory and helper cells have memory-like properties. J Clin Invest.

[49] Laidlaw BJ (2017). Interleukin-10 from CD4^+^ follicular regulatory T cells promotes the germinal center response. Sci Immunol.

[50] Jouvenne P (1998). Elevated levels of soluble interleukin-1 receptor type II and interleukin-1 receptor antagonist in patients with chronic arthritis: correlations with markers of inflammation and joint destruction. Arthritis Rheum.

[51] Hao Y (2024). Dictionary learning for integrative, multimodal and scalable single-cell analysis. Nat Biotechnol.

[52] Street K (2018). Slingshot: cell lineage and pseudotime inference for single-cell transcriptomics. BMC Genomics.

[53] Lorenzon R (2018). Clinical and multi-omics cross-phenotyping of patients with autoimmune and autoinflammatory diseases: the observational TRANSIMMUNOM protocol. BMJ Open.

[54] Pitoiset F (2018). A standardized flow cytometry procedure for the monitoring of regulatory T cells in clinical trials. Cytometry B Clin Cytom.

[55] Pitoiset F (2018). Deep phenotyping of immune cell populations by optimized and standardized flow cytometry analyses. Cytometry A.

[56] Tchitchek N (2024). Deep immunophenotyping reveals that autoimmune and autoinflammatory disorders are spread along two immunological axes capturing disease inflammation levels and types. Ann Rheum Dis.

