# Supplementary material for: IL-1β signaling modulates T follicular helper and regulatory cells in human lymphoid tissues
Source: JCI Insight. 2025 May 20;10(12):e188724. doi: 10.1172/jci.insight.188724 (PMC12220957; doi:10.1172/jci.insight.188724)
Supplement: Supplemental data [file jciinsight-10-188724-s153.pdf]

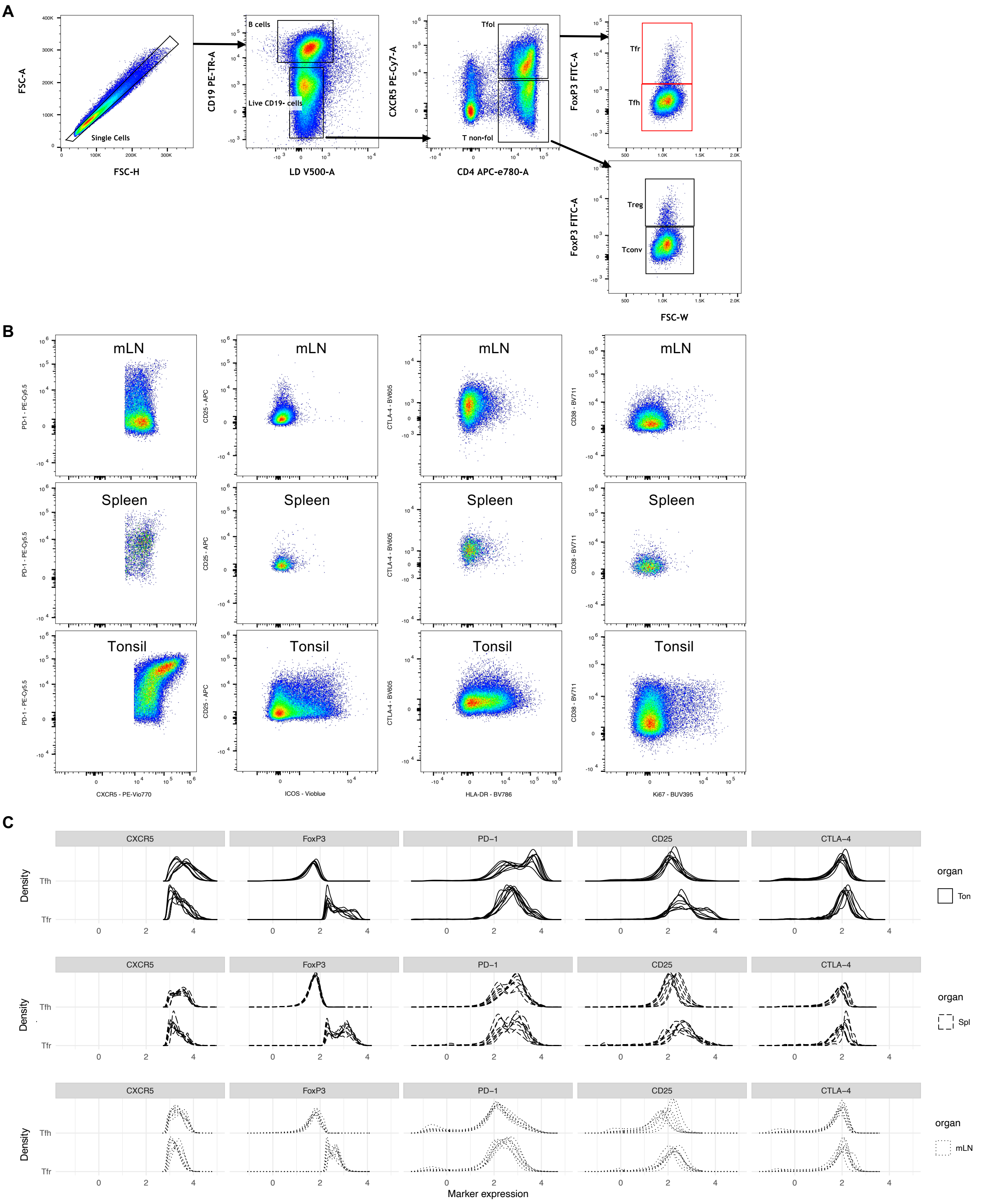

Figure S1. Identification of Tfh and Tfr and their marker expression patterns.
(**A**) Flow cytometry gating strategy to identify follicular T cell (Tfol) subsets (Tfh and Tfr), as well as non-follicular T cell subsets (Tconv and Treg). (**B**) Illustrations of flow cytometry staining within Tfh from mLNs (top), spleen (middle) and tonsils (bottom) for markers CXCR5, PD-1, ICOS, CD25, HLA-DR, CTLA-4, Ki-67 and CD38. (**C**) Density plots showing marker expression as logicle-transformed MFI per sample in Tfh (top) and Tfr (bottom) across tonsils (Ton), Spleens (Spl), and mesenteric lymph nodes (mLN) (n=8 each).

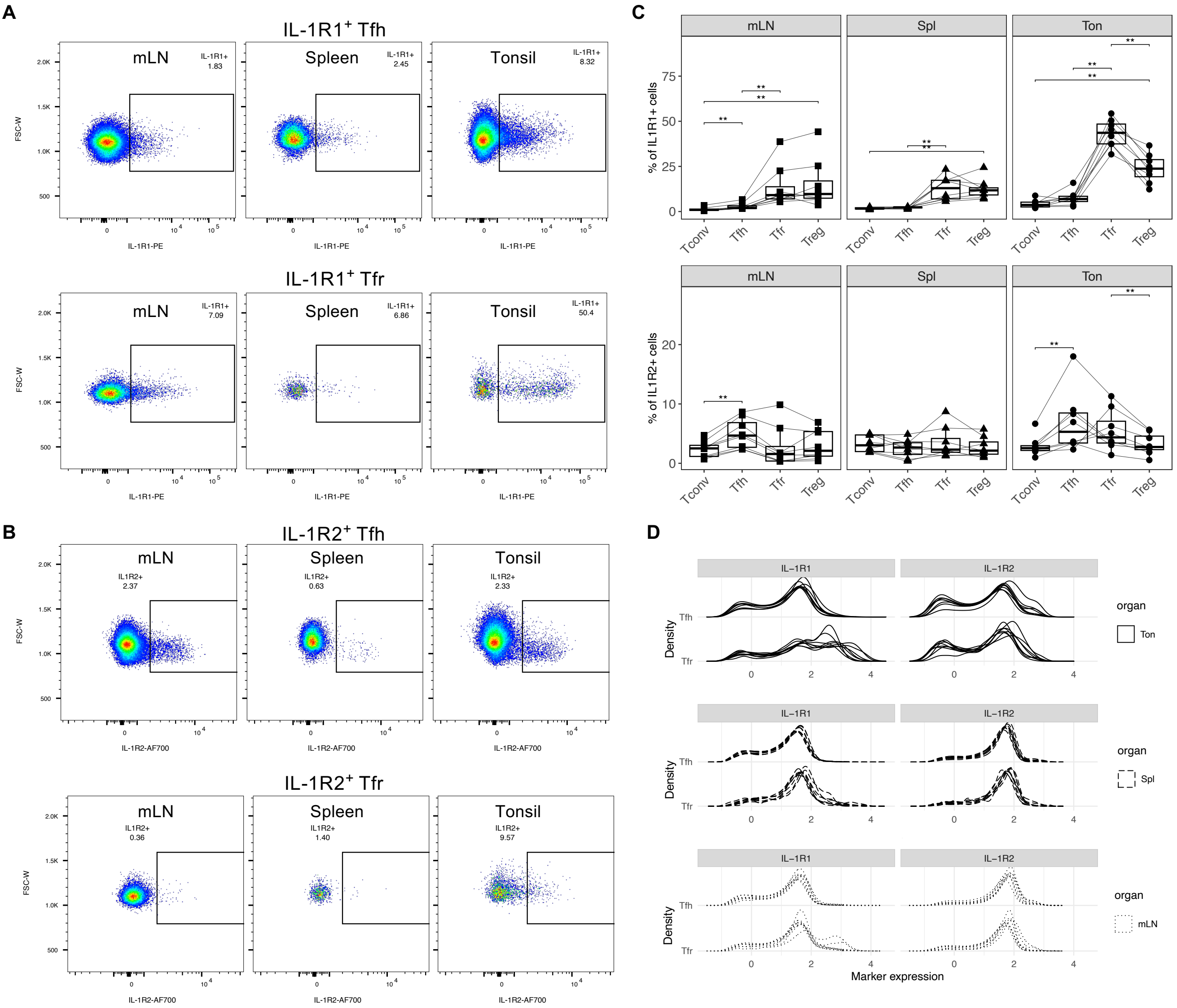

Figure S2. Comparison of IL-1R1 and IL-1R2 expression between follicular and non-follicular T cell subsets within lymphoid organs.

(**A**) Example of IL-1R1⁺ flow cytometry staining among Tfh (top) and Tfr (bottom), within mLNs, spleen and tonsils. (**B**) Illustrations of IL-1R2⁺ flow cytometry staining among Tfh (top) and Tfr (bottom), within mLNs, spleen and tonsils. (**C**) Percentage of IL-1R1⁺ (top) and IL-1R2⁺ (bottom) cells among CD4⁺ T cell subsets. (**D**) Density plots showing IL-1R1 and IL-1R2 logicle-transformed expression per sample in Tfh (top) and Tfr (bottom) across tonsils (Ton), Spleens (Spl), and mesenteric lymph nodes (mLN) (n=8 each). **P < 0.01 by paired Wilcoxon test.

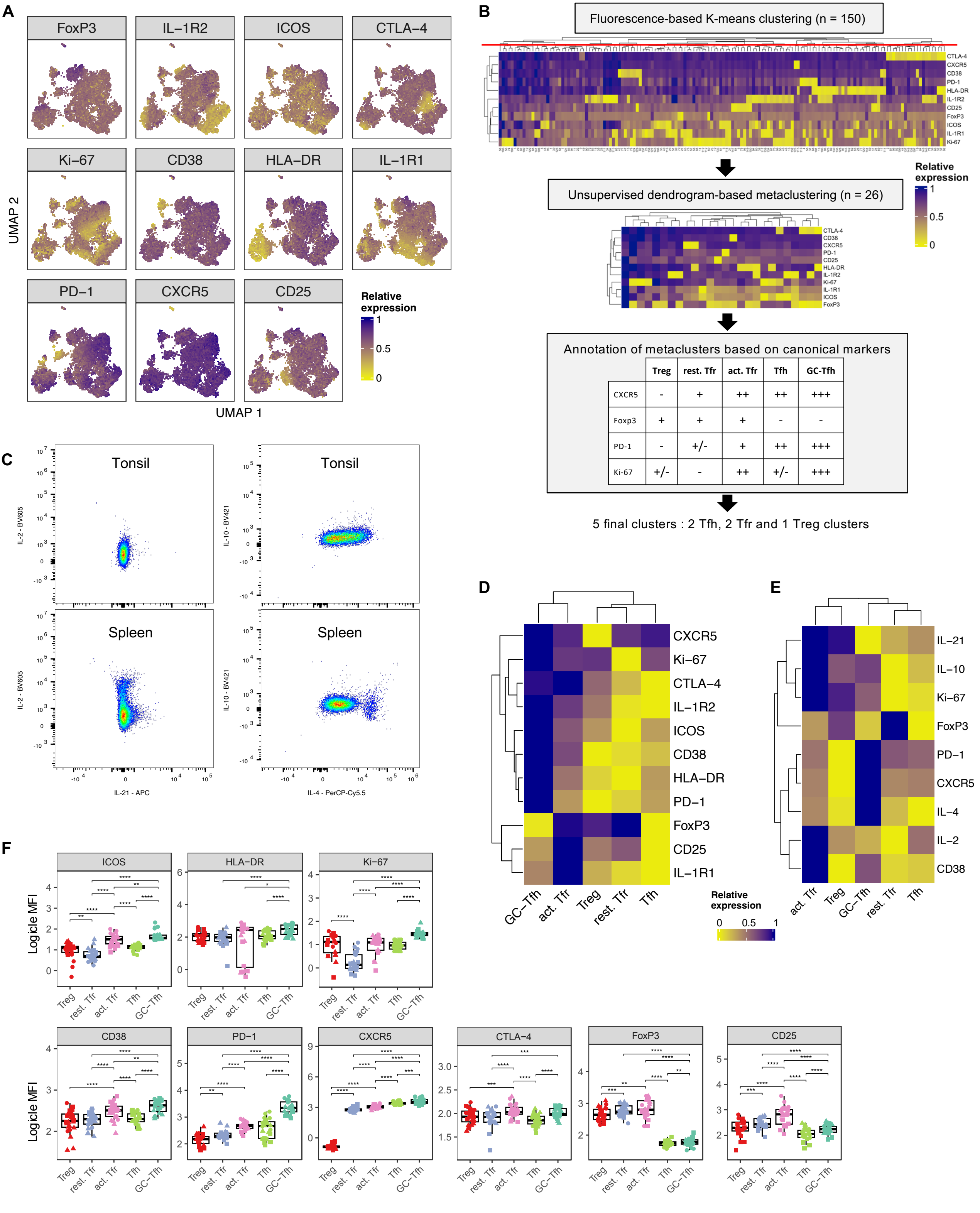

Figure S3. Identification of Tfh, Tfr, and Treg clusters across human secondary lymphoid organs.
(**A**) UMAP representations of CD4⁺ CXCR5⁺ (Tfh and Tfr) cells and CXCR5⁻ Foxp3⁺ cells (Treg), overlayed by the expression of the different cell markers. (**B**) Analysis strategy used for the annotation of follicular T cell and Treg clusters. (**C**) Illustrations of flow cytometry staining for IL-21, IL-2, IL-10 and IL-4 within Tfol from a tonsil (top) and a spleen (bottom). (**D-E**) Heatmap showing relative marker expression of the 5 identified cell clusters with the phenotypic panel (**D**) and the functional panel (**E**). (**F**) Logicle-MFI of markers used for clustering across the 5 clusters, among all organs. *P < 0.05; **P < 0.01; ***P < 0.001; ****P < 0.0001 by Mann-Whitney test.

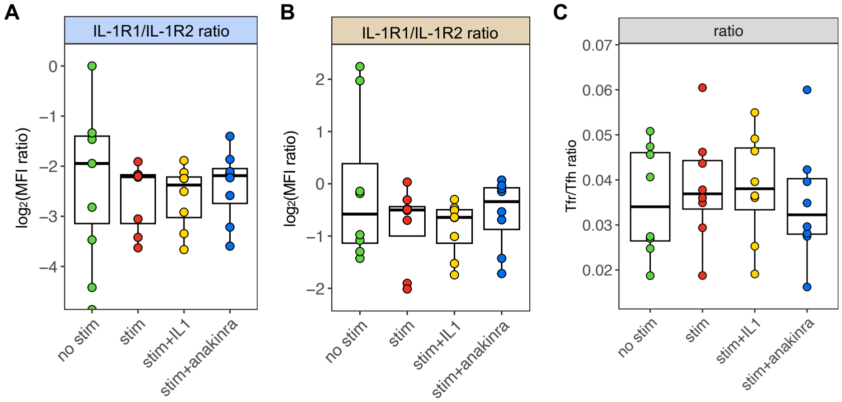

Figure S4. Modulation of IL-1Rs and IL-1β signaling during in vitro Tfh and Tfr stimulation.

(**A-B**) Boxplots showing the ratio IL-1R1 and IL-1R2 MFI among Tfh (**A**) and Tfr (**B**) from whole-cell tonsillar cultures compared to no stimulation (n = 8). (**C**) In vitro Tfr/Tfh cell ratio within each condition.

A

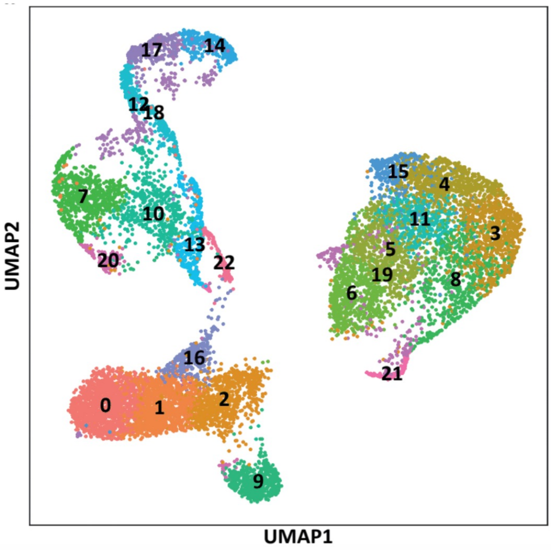

#cluster	annotation
0	GC-Tfh
1	act. Tfr
2	act. Tfr
3	act. Tfr
4	Tconv
5	act. Tfr
6	act. Tfr
7	Tfh
8	GC-Tfh
9	GC-Tfh
10	rest. Tfr
11	Tfh
12	Tconv
13	Tfh
14	Tconv
15	Tconv
16	GC-Tfh
17	Tconv
18	Tconv
19	act. Tfr
20	Tfh
21	act. Tfr
22	Tfh

B

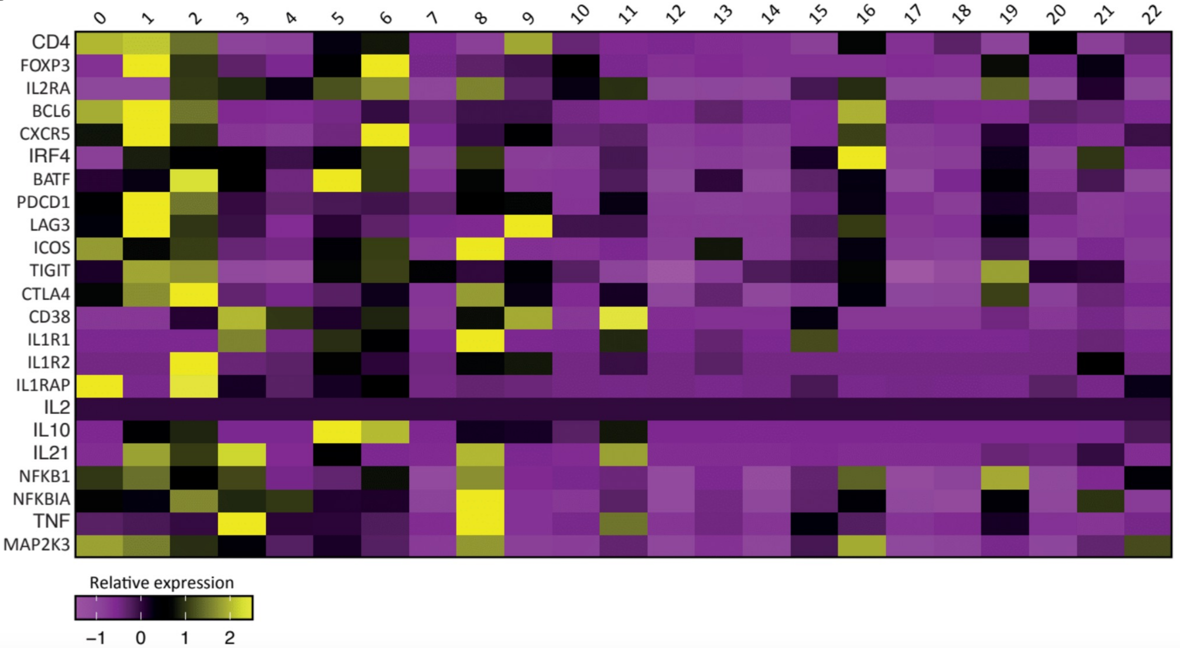

Figure S5. Cluster identification and characterization from tonsillar single-cell transcriptomics.
(**A**) UMAP representation showing the identified clusters with their label identifier (#cluster) and associated biological annotations. (**B**) Heatmap representation showing the relative expression of selected genes in the different clusters.

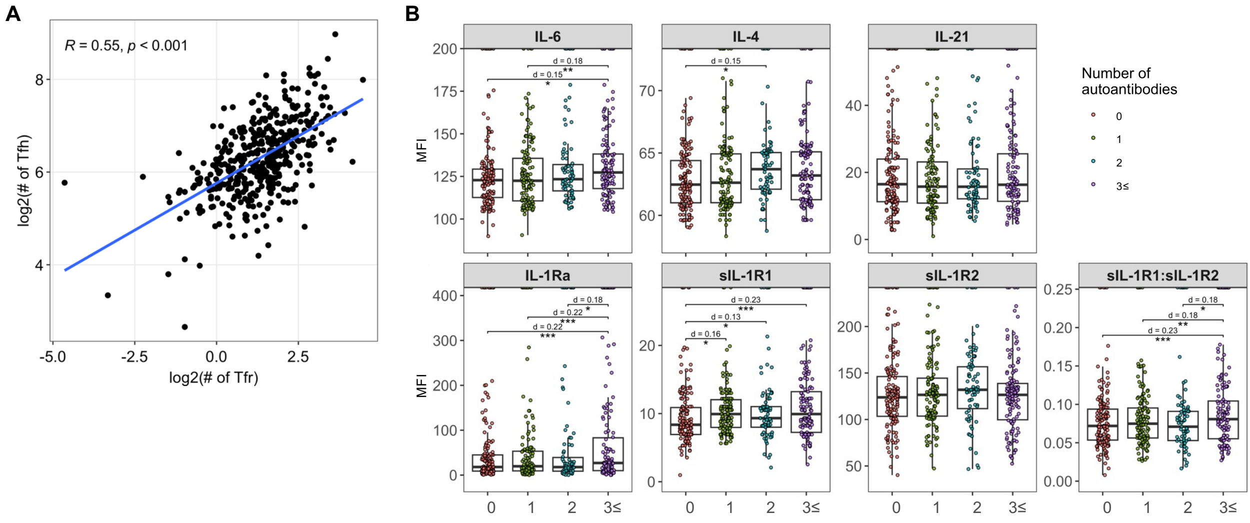

Figure S6. Correlations between circulating Tfh, Tfr and increase of IL-1β-related molecules within individuals of the Trans*i*mmunom cohort.

(**A**) Scatter plot showing the relationship between the number of cTfr and cTfh in the Transimmunom cohort, with the Spearman correlation coefficient and the corresponding p-value. (log2-transformed). (**B**) MFI measurements of peripheral cytokine and IL-1 related molecules according to the number of autoantibodies in individuals. *P < 0.05; **P < 0.01; ***P < 0.001; ****P < 0.0001 by Mann-Withney test.

Supplemental Table 1. List of the selected 154 genes involved in the regulation of IL-1β

Gene name		
MYD88	MAPK3	MAPKAPK2
IRAK4	MAPK4	IKBKE
IRAK1	MAPK6	RBX1
IRAK2	MAPK7	NOD1
TRAF6	MAPK8	TAB1
CXCL8	MAPK11	UBE2E3
CCL2	MAPK9	MAP3K2
PTGS2	MAPK10	UBE2C
NFKBIA	MAPK13	IRAK3
IL1R1	MAP2K1	ERC1
IL1R2	MAP2K2	TAB2
IL1RN	MAP2K3	UBE2S
IL1B	MAP2K6	UBE2T
IL1A	MAP2K7	IRAK4
IL1RAP	PTGS2	ECSIT
CASP1	PTPN11	UBE2J1
CHUK	REL	UBE2D4
MAP3K8	RELA	TOLLIP
ATF2	MAPK12	UBE2R2
MAPK14	CCL2	UBE2W
DUSP1	MAP2K4	UBE2Q1
ELK1	SKP1	PELI2
ELK4	MAP3K7	PELI1
FOS	TNFAIP3	UBE2O
UBE2K	TRAF6	NOD2
HSPB1	UBE2A	UBE2Z
HSPB2	UBE2B	TNIP2
IKBKB	UBE2D1	IL1F10
IL1A	UBE2D2	UBE2Q2
IL1B	UBE2D3	UBE2J2
IL1R1	UBE2E1	UBE2F
IL1RAP	UBE2E2	UBE2U
IL1RN	UBE2G1	MAPK15
IL6	UBE2G2	PELI3
CXCL8	UBE2H	TAB3
IRAK1	UBE2I	TICAM2
IRAK2	UBE2L3	UBE2L2
JUN	UBE2N	AKT1
MAP3K1	UBE2V1	CASP1
MAP3K3	UBE2V2	IL1A
MYD88	ZFP36	IL1B
NFKB1	IL1R2	IL18
NFKBIA	CUL1	NFKB1
NFKBIB	IKBKG	NFKB2
PIK3CA	KHSRP	RELA
PIK3R1	RIPK2	STS
PIK3R2	SQSTM1	CASP1
PLCG1	BTRC	IL1B
PRKCI	MAP3K14	P2RX7
PRKCZ	UBE2M	ATP8A2
MAPK1	UBE2L6	NLRP3

Supplemental Table 2.Techniques for detecting and quantifying autoanticoprs in the Transimmunom cohort

Acronyme	Names	Technique	Commercial Kit
ACL IgG	Anti-cardiolipin IgG antibodies	Multiplex Fow Immunoassay	BioPlex2200 (Biorad)
ACL IgM	Anti-cardiolipin IgM antibodies	Multiplex Fow Immunoassay	BioPlex2200 (Biorad)
ANCA	Anti-neutrophil cytoplasm antibodies	Indirect immunofluorescence assay (Neutophils)	EuroImmun
Anti Znt8	Anti-Zinc Transporter 8 antibodies	ELISA	Theradiag
anti-CCP	Anti-cyclic citrullinated peptide antibodies	ELISA	Quanta lite INOVA
anti-centromere	Anti-centromere antibodies	Multiplex Fow Immunoassay	BioPlex2200 (Biorad)
anti-EJ	Anti-glycyl-tRNA synthetase antibodies	Dots	EuroImmun
Anti-GAD65	Anti-glutamic acid decarboxylase antibodies	ELISA	Theradiag
anti-gp210	Anti-glycoprotein-210 antibodies	ELISA	Quanta lite INOVA
anti-HmGCoA	Anti-3-hydroxy-3-méthylglutaryl-coenzyme A antibodies	ELISA	Quanta lite IL
Anti-IA2	Anti-tyrosine phosphatase antibodies	ELISA	Theradiag
Anti-insulin	Anti-Insulin antibodies	ELISA	Theradiag
anti-JO1	Anti-Jo1 antibodies	Multiplex Fow Immunoassay	BioPlex2200 (Biorad)
anti-KU	Anti-Ku antibodies	Dots	EuroImmun
anti-MDA5	Anti-melanoma differentiation-associated protein 5 antibodies	Dots	EuroImmun
anti-Mi2	Anti-Mi2 antibodies	Dots	EuroImmun
anti-MPO	Anti-myeloperoxidase antibodies	ELISA	EuroImmun
anti-nDNA	Anti-native DNA antibodies	Farr assay (RIA)	Amerlex
ANA	Antinuclear antibodies	Indirect immunofluorescence assay (Hep-2)	EuroImmun
anti-NXP2	Anti-NXP2 antibodies	Dots	EuroImmun
anti-OJ	Anti-isoleucyl-tRNA synthetase antibodies	Dots	EuroImmun
anti-PL12	anti-alanyl-tRNA synthetase antibodies	Dots	Dtech
anti-PL7	Anti-threonyl-tRNA synthetase antibodies	Dots	Dtech
anti-PLA2R	Anti-phospholipase A2 receptor antibodies	ELISA	EuroImmun
anti-PmScl	Anti-PM/Scl antibodies	Dots	EuroImmun
anti-PR3	Anti-proteinase-3 antibodies	ELISA	EuroImmun
anti-ribosome	Anti-ribosomal antibodies	Multiplex Fow Immunoassay	BioPlex2200 (Biorad)
anti-RNP	Anti-ribonucleoprotein antibodies	Multiplex Fow Immunoassay	BioPlex2200 (Biorad)
anti-Ro52	Anti-Ro52 antibodies	Multiplex Fow Immunoassay	BioPlex2200 (Biorad)
anti-SAE1	Anti-SUMO1 Activating Enzyme Subunit 1 antibodies	Dots	EuroImmun
anti-Scl70	Anti-Scl70 antibodies	Multiplex Fow Immunoassay	BioPlex2200 (Biorad)
anti-Sm	Anti-Smith antibodies	Multiplex Fow Immunoassay	BioPlex2200 (Biorad)
anti-sp100	Anti-sp100 antibodies	ELISA	Quanta lite INOVA
anti-SRP	Anti-signal recognition particle antibodies	Dots	Dtech
anti-SSA60	Anti-SSA60 antibodies	Multiplex Fow Immunoassay	BioPlex2200 (Biorad)
anti-SSB	Anti-Sjögren's syndrome type B antibodies	Multiplex Fow Immunoassay	BioPlex2200 (Biorad)
anti-TIF1γ	Anti-transcription intermediary factor 1-gamma antibodies	Dots	EuroImmun
anti-β2GP1 IgG	Anti-Beta-2 glycoprotein 1 IgG antibodies	Multiplex Fow Immunoassay	BioPlex2200 (Biorad)
anti-β2GP1 IgM	Anti-Beta-2 glycoprotein 1 IgM antibodies	Multiplex Fow Immunoassay	BioPlex2200 (Biorad)
ASCA IgA	Anti-Saccharomyces cerevisiae IgA antibodies	Indirect immunofluorescence assay (Sacc. Cerevisia)	EuroImmun
ASCA IgG	Anti-Saccharomyces cerevisiae IgG antibodies	Indirect immunofluorescence assay (Sacc. Cerevisia)	EuroImmun
ENA	Extractable nuclear antigens	Dots	EuroImmun
Latex	Latex	Agglutination	FUMOUZE
GMB	Anti-glomerular basement membrane	Indirect immunofluorescence assay	EuroImmun
Waaler rose	Waaler rose	Hemagglutination	Biomérieux
